# Glycoursodeoxycholic acid ameliorates diet-induced metabolic disorders with inhibiting endoplasmic reticulum stress

**DOI:** 10.1042/CS20210198

**Published:** 2021-07-20

**Authors:** Lele Cheng, Tao Chen, Manyun Guo, Peining Liu, Xiangrui Qiao, Yuanyuan Wei, Jianqing She, Bolin Li, Wen Xi, Juan Zhou, Zuyi Yuan, Yue Wu, Junhui Liu

**Affiliations:** 1 Department of Cardiovascular Medicine, The First Affiliated Hospital of Xi’an Jiaotong University, Xi’an, Shaanxi, China; 2 Department of Medical Imaging, The First Affiliated Hospital of Xi’an Jiaotong University, Xi’an, Shaanxi, China; 3 Key Laboratory of Environment and Genes Related to Diseases, Ministry of Education, Xi’an, Shaanxi, China; 4 Key Laboratory of Molecular Cardiology, Shaanxi Province, Xi’an, Shaanxi, China; 5 Clinical Laboratory, The First Affiliated Hospital of Xi’an Jiaotong University, Xi’an, Shaanxi, China

**Keywords:** Bile acids, Endoplasmic reticulum stress, Glycoursodeoxycholic acid, Metabolic disorders

## Abstract

Recent studies reveal that bile acid metabolite composition and its metabolism are changed in metabolic disorders, such as obesity, type 2 diabetes and metabolic associated fatty liver disease (MAFLD), yet its role and the mechanism remain largely unknown. In the present study, metabolomic analysis of 163 serum and stool samples of our metabolic disease cohort was performed, and we identified glycoursodeoxycholic acid (GUDCA), glycine-conjugated bile acid produced from intestinal bacteria, was decreased in both serum and stool samples from patients with hyperglycemia. RNA-sequencing and quantitative PCR results indicated that GUDCA alleviated endoplasmic reticulum (ER) stress in livers of high fat diet (HFD)-fed mice without alteration of liver metabolism. *In vitro*, GUDCA reduced palmitic acid induced-ER stress and -apoptosis, as well as stabilized calcium homeostasis. *In vivo*, GUDCA exerted effects on amelioration of HFD-induced insulin resistance and hepatic steatosis. In parallel, ER stress and apoptosis were decreased in GUDCA-treated mice as compared with vehicle-treated mice in liver. These findings demonstrate that reduced GUDCA is an indicator of hyperglycemia. Supplementation of GUDCA could be an option for the treatment of diet-induced metabolic disorders, including insulin resistance and hepatic steatosis, with inhibiting ER stress.

## Introduction

Metabolic disorders including type 2 diabetes mellitus (T2DM) and metabolic associated fatty liver disease (MAFLD), which is suggested to replace the current name of nonalcoholic fatty liver disease (NAFLD) as a more appropriate overarching term [1]. T2DM and MAFLD are global health threats with rapid increase of prevalence over the past two decades [2–4]. Studies demonstrate a strong association among obesity, T2DM and MAFLD that more than 90% of obese patients with T2DM have MAFLD [5], and increase the risk for progression of hepatic steatosis to steatohepatitis and end-stage liver diseases such as cirrhosis and hepatocellular carcinoma [6]. Obesity with T2DM and MAFLD increases the risk for cardiovascular-related morbidity and mortality [7,8]. In order to develop effective interventions of T2DM and MAFLD, a comprehensive understanding of the mechanism of pathogenesis of metabolic disorders is urgently needed.

Accumulating evidences indicate that the gut microbiota influences the development of insulin resistance and hepatic steatosis [9–11]. The gut microbiota influences the host metabolism via producing the lipopolysaccharides, secondary bile acids (BAs), short chain fatty acids (SCFAs) and trimethylamine N-oxide (TMAO) [12]. SCFAs, comprising mainly acetate, propionate and butyrate, have been well described to regulate host energy metabolism [13–15]. TMAO, a toxic compound derived from choline by gut bacteria, contributes to the onset of atherosclerosis in rodent models and is strongly associated with cardiovascular disease risk [16]. Our previous data demonstrated that metformin increases the levels of conjugated BAs in the gut by reducing the abundance of *Bacteroides fragilis* species to antagonize intestinal farnesoid X receptor (FXR)-fibroblast growth factor 19 (FGF19) signaling, thereby improving insulin sensitivity in T2DM patients [17]. Therefore, it is of great clinical importance to study the role of the secondary BAs in the pathogenesis of T2DM and MAFLD.

BAs are produced from cholesterol by hepatocytes, stored in the gall bladder and then released into the gut after a meal. BAs facilitate absorption of triglycerides, cholesterol and lipid-soluble vitamins [18]. The primary BAs, cholic acid (CA) and chenodeoxycholic acid (CDCA), are metabolized by the gut microbiota to generate the secondary BAs, deoxycholic acid (DCA) and lithocholic acid (LCA), respectively [18]. In mouse liver, CDCA is converted to α- and β-muricholic acids(α/βMCA), while in human CDCA can be converted to ursodeoxycholic acid (UDCA) by gut microbial 7α/β-dehydrogenation. BAs are conjugated to glycine or taurine to form conjugated BAs for biliary secretion [18]. Total BA concentrations are increased in obese patients and correlate with body mass index irrespective of T2DM and MAFLD [19]. BAs are metabolic sensors that active FXR signaling to control lipid, glucose and energy metabolism [17,20]. The endoplasmic reticulum (ER) is the subcellular compartment involved in protein synthesis, folding and transporting, lipid biosynthesis and calcium homeostasis. Dysregulation of ER is a key pathogenic factor that causes insulin resistance and hepatic steatosis in metabolic disorders [21]. Tauroursodeoxycholic acid (TUDCA) enhances the adaptive capacity of ER and acts as a potent anti-diabetic modality [22].

BA signaling plays a critical role in regulation of metabolic homeostasis. The systemic changes of the BA metabolite profile in patients with metabolic disorders and the detailed mechanism remain elusive. To fill this gap, we sought to identify endogenous BA metabolic regulators via analysis of BA metabolite profile by targeted metabolomics in patients with glucose metabolic disorders and explore the underlying mechanism. Herein, we found that glycoursodeoxycholic acid (GUDCA) was reduced markedly in patients with hyperglycemia. Supplementation of GUDCA alleviated high fat diet (HFD)-induced insulin resistance and hepatic steatosis and decreased ER stress and apoptosis in mice. This work suggests that GUDCA supplementation may be a therapeutic option for patients with diet-induced metabolic disorders.

## Materials and methods

### Human sample collection

We collected serum and stool samples from patients of our metabolic cohort in the First Affiliated Hospital of Xi’an Jiaotong University, Xi’an, China. The patients did not have gastrointestinal diseases, biliary tract diseases, severe diabetic complications, severe hepatic diseases, a special diet or pregnancy. All participants signed the written informed consent form before sample collection. Clinical parameters were determined at the First Affiliated Hospital of Xi’an Jiaotong University. The basic characteristics are listed in Table 1. The study protocol was approved by the ethnic committee of the First Affiliated Hospital of Xi’an Jiaotong University.

**Table 1:** Baseline characteristics of the subjects

Characteristics	Low (*n*=135)	High (*n*=28)	*P* value
Gender, *n* (%)			a
Male	84 (62.2%)	18 (64.3%)	
Female	51 (37.8%)	10 (35.7%)	
Age (y)	59.23 ± 0.90	60.40 ± 1.52	0.581
History of hypertension (%)	62 (45.9%)	13 (46.4%)	0.658
History of smoking (%)	85 (62.9%)	16 (57.1%)	0.168
History of drinking (%)	105 (77.7%)	21 (75.0%)	0.845
Body mass index (kg/m^2^)	24.94 ± 0.32	26.53 ± 0.93	0.058
HbA1c (%)	7.72 ± 0.22	5.52 ± 0.04	<0.001
Fasting glycaemia (mmol/l)	7.30 ± 0.13	5.00 ±0.06	<0.001
ALT (U/l)	25.39 ± 1.28	26.65 ± 2.47	0.685
AST (U/l)	25.24 ± 2.76	27.52 ± 6.25	0.737
TG (mmol/l)	1.63 ± 0.10	2.29 ± 0.34	0.073
TC (mmol/l)	4.03 ± 0.09	3.85 ± 0.18	0.454
HDL-C (mmol/l)	1.05 ± 0.02	0.93 ± 0.05	0.036
LDL-C (mmol/l)	2.36 ± 0.08	2.17 ± 0.14	0.524

Abbreviations: ALT, alanine aminotransferase; AST, aspartate aminotransferase; HbA1c, hemoglobin A1c; HDL-C, high-density lipoprotein cholesterol; LDL-C, low-density lipoprotein cholesterol; TC, total cholesterol; TG, triglyceride. “a” represents *P* value = 1.

### Bile acid profiles measurement

BA concentrations were determined using a UPLC-tandem MS method as described previously [17,23]. In brief, for the quantitative profiling of BAs, an aliquot of 50 µl of plasma was mixed with 300 µl of ice-cold methanol (with 0.01% ammonium hydroxide) and 10 µl of internal standard. Feces (10 mg) homogenized with 100 μl of water at 3000 rpm using homogenizer. Ten microliters of internal standard and 300 μl ice-cold methanol (with 0.01% ammonium hydroxide) were added for extraction BAs. All mixtures were allowed to stand at 4°C for 10 min and vortexed for 45 s, and then centrifuged at 16000 × ***g*** for 10 min at 4°C. The supernatant was transferred to an autosampler vial and 5 µl was injected for LC-MS analysis. Chlorpropamide (Sigma-Aldrich, U.S.A.) was applied as an internal standard to quantify the BAs. Targeted analysis of BA was performed using UPLC/Synapt G2-Si QTOF MS system (Waters Corp., Milford, MA, U.S.A.) with an ESI source. An Acquity C18 BEH column (2.1 mm × 100 mm, 1.7 μm, Waters Corp.) was maintained at 45°C and a flow rate of 0.4 ml/min was used for chromatographic separation with a gradient of mixing of the mobile phases 0.1% formic acid in water and 0.1% formic acid in acetonitrile. MS was detected in a negative ionization mode. A mass range of *m/z* 50–850 was acquired. External standards for all BAs were used to identify the different BA metabolites, including CA, α-MCA, β-MCA, dehydrocholic acid (DHCA), taurocholic acid (TCA), UDCA, hyodeoxycholic acid (HDCA), taurochenodeoxycholic acid (TCDCA), taurodeoxycholic acid (TDCA), taurolithocholic acid (TLCA), glycocholic acid (GCA), glycolithocholic acid (GLCA), GUDCA, glycochenodeoxycholic acid (GCDCA), glycodeoxycholic acid (GDCA), CDCA, DCA, LCA, tauro-β-muricholic acid (TβMCA), TUDCA and taurohyodeoxycholic acid (THDCA).

### Chemicals and reagents

GUDCA, TUDCA, Sodium carboxymethyl cellulose (CMC-Na) and PluronicF-127 were purchased from Sigma-Aldrich (St. Louis, MO, U.S.A.). Antibody against Phospho-IRE(Ser724) was supplied by Abcam (Cambridge, MA, U.S.A.). Phosphorylated protein kinase-like ER kinase (p-PERK Thr980) was ordered from by Santa Cruz Biotechnology (Santa Cruz, CA, U.S.A.). The antibodies against Phospho-p38MAPK (Thr180/Tyr182), Phospho-JNK (Thr183/Tyr185), CHOP, Phospho-eIF2α (Ser51), Cleaved-caspase3, Phospho-Akt (Ser473) and Irs1 were all purchased from Cell Signaling Technology (Danvers, MA, U.S.A.). Antibodies against β-actin, Tubulin, GAPDH, Bax and Bcl2 were obtained from Proteintech Group (Wuhan, China). Horseradish peroxidase conjugated goat anti-rabbit and anti-mouse antibodies were obtained from Proteintech Group (Wuhan, China). Fluo-3/AM and Fura-Red were purchased from Thremo Fisher Scientific (Molecular Probes Inc, U.S.A.). All solvents and organic reagents were of the highest grade available.

### Animal models

Experimental protocols in our study were approved by the Institutional Ethics Committee for Animal Experiments of Xi’an Jiaotong University. All procedures conformed to the U.S. National Institutes of Health Guide for the Care and Use of Laboratory Animals. The manipulation of animals were performed in the Animal Center of Xi’an Jiaotong University. All rodents were male and maintained in Specific Pathogen Free (SPF) environment. Mice aged 5 to 6 weeks were used and were maintained under a 12 h light/12 h dark cycle at 23 ± 1°C and humidity (45–55%) with free access to food and water. To explore the mechanism of GUDCA on metabolism by RNA-Sequencing, mice were provided with HFD (Research Diets, #D12109C, U.S.A.) +vehicle (CMC-Na) or HFD+GUDCA (80 mg/kg/d) for 1 week. In addition, HFD was fed to mice for 8 weeks to induce diet-related metabolic dysfunction. During the last 3 weeks, HFD-fed mice were randomly treated with 80 mg/kg/d of GUDCA or 350 mg/kg/d of TUDCA or vehicle by intraperitoneal injection. After treatment, the mice were humanely killed, no anesthetic was used. The serum samples and some tissues were harvested and fixed in paraffin or stored at −80°C.

### RNA sequencing

Mouse liver RNAs were extracted in accordance with the manual of TRIzol ® (Life Technologies, Inc, U.S.A.). Preparation of library and sequencing of transcriptome were carried out using Illumina NovaSeq 6000 (Illumina, U.S.A.). Clean data were obtained by removing reads containing adapters, reads containing poly-N and low-quality reads from raw data. The high-quality paired-end reads from each library were mapped to mus mouse’s reference genome (http://genome.ucsc.edu/cgi-bin/hgGateway?db=mm10) using hisat2 v.2.1.0. The transcripts were calculated and normalized to FPKM (fragments per kilobase of transcript sequence per million mapped reads), representing the gene expression levels. DESeq software was used to identify differentially expressed genes (DEGs) in pair-wise comparisons. Genes with an adjusted *P*-value <0.05 found by DESeq were assigned as differentially expressed ones. Gene Ontology (GO) enrichment analysis of DEGs was implemented by the GOseq R package in which gene length bias was corrected (http://www.geneontology.org/) [24]. Kyoto Encyclopedia of Genes and Genomes (KEGG, http://www.genome.jp/kegg/) analysis was performed using clusterProfiler R package [25].

### RNA isolation and real-time quantitative PCR analysis

Total RNA was extracted from cultured cells using RNAprep Pure cell/Bacteria Kit (DP430) (TIANGEN, China) according to the manufacturer’s instructions. All tissue samples were stored in liquid nitrogen immediately after mice sacrifice and then moved to −80°C. The TRIzol reagent was used to isolate total RNA from the frozen tissue with a standard phenol-chloroform extraction protocol. Purified RNA was reverse-transcribed into cDNA by using a Revert Aid First Strand cDNA Synthesis Kit (Thermo Fisher Scientific, Waltham, MA, U.S.A.), and newly synthesized cDNA was analyzed by real-time quantitative PCR on iQ5 Multicolor Real-Time PCR detection system (Bio-Rad, Hercules, CA, U.S.A.) with a SYBR Premix Ex TaqII RT-PCR Kit (Takara, Kyoto, Japan). The real-time PCR primer sequences are listed in Supplementary Table S1. The relative abundances of the involved genes were calculated by normalizing to GAPDH.

### Cell culture

HepG2 cells were purchased from the American Type Culture Collection (Manassas, VA, U.S.A.). Cells were cultured in DMEM (Thermo Fisher Scientific, Waltham, MA, U.S.A.) supplemented with 10% fetal bovine serum (Thermo Fisher Scientific, Waltham, MA, U.S.A.) and maintained at 37°C in a humidified atmosphere of 5% CO_2_. Cells were seeded in a six-well plate and incubated in serum-free medium overnight and then exposed to the indicated concentrations of different interventions.

### Immunoblotting

Cultured cells were lysed in RIPA buffer (Cybrdi, Gaithersburg, MD, U.S.A.) that contained protease inhibitor (Roche Diagnostics, Indianapolis, IN, U.S.A.). Protein concentration was determined using BCA protein assay reagent kit (Thermo Fisher Scientific, Waltham, MA, U.S.A.). Equal amounts of protein were loaded on to 8–12% Bis-Tris precast gels for electrophoresis and were electrotransferred onto a PVDF membrane (Roche Life Science, Basel, Switzerland). After blocking for 1 h at room temperature, membranes were sequentially incubated with primary antibodies at 4°C overnight and secondary antibodies at room temperature for 1 h. Immunoreactive bands were visualized using ECL substrate in a ChemiDoc XRS Imaging system (Bio-Rad, U.S.A.). Target proteins were normalized to β-actin, Tubulin or GAPDH, respectively.

### Fatty acid uptake assays

Boron-dipyrromethene (BODIPY)-C16 was purchased from Invitrogen Life Sciences (California, U.S.A.). Stock solution of BODIPY-C16 (2 mM) was prepared in DMSO. HepG2 cells were seeded in 24-well plates at an appropriate density cells per well with serum-free DMEM for 24 h. Following GUDCA (300 μM) treatment, cells were incubated in culture medium containing 2 μM BODIPY-C16 for 30 min at 37°C. After washing with PBS three times, then fixed in ice-cold 4% paraformaldehyde for another 10 min. The fluorescence was measured using an EnSpire multimode plate reader (PerkinElmer, Waltham, MA, U.S.A.; excitation 500 nm and emission 515 nm) and inverted fluorescence microscope (Olympus, Tokyo, Japan).

### Annexin V/propidium iodide staining

Annexin V/propidium iodide (PI) assay was performed according to the manufacturer instructions (Annexin V-FITC kit; Roche Life Sciences, Basel, Switzerland). In brief, cells were washed with PBS, then incubated with the mixture of Annexin V and PI at room temperature for 10 min in the dark. The fluorescence signal was detected by using a fluorescence microscope (Olympus, Tokyo, Japan). Photomicrographs were obtained using identical exposure settings, and all images were quantified using Image-Pro Plus 6.0 software (Media Cybernetics, Rockville, MD, U.S.A.).

### TUNEL labeling

Cell death in situ examination was conducted using the In Situ TUNEL Kit (Roche Life Sciences, Basel, Switzerland) according to the manufacturer’s instructions. In brief, cell-attached slides or tissue sections were sequentially incubated with TUNEL reaction mixture at 37°C in a humidified chamber for 60 min. Images were obtained using identical exposure settings and were quantified by using Image-Pro Plus 6.0 software.

### Intracellular calcium measurement

Intracellular calcium concentration was measured by confocal microscopy using Fluo-3/Fura-Red fluorescence. Cells pretreated with 300 μM GUDCA or vehicle were incubated with 5 μM Fluo-3 and 10 μM Fura-Red with 0.02% pluronic F-127 at 37°C for 1 h. Palmitate (PA) at a final concentration of 200 μM was added immediately before analysis at 488 nm excitation. A 530 nm filter and a 605 nm filter were used to separate the emitted light from each other (Leica, TCS, SP8, Germany). The ratio of Fluo-3 fluorescence to Fura-Red fluorescence was calculated to reflect cytoplasmic calcium concentration.

### Metabolic assays

Glucose tolerance tests (GTT) were performed after 16–18 h fasting. Blood glucose concentrations were measured with a blood glucose test meter and blood samples were taken from the tail tip at 0, 15, 30, 60 and 120 min after intraperitoneal injection of glucose (1.5 g/kg body weight). For the insulin tolerance test (ITT), insulin (0.5 U/kg body weight) was administered via intraperitoneal injection after 4 h fasting.

### H&E staining

Paraffin-embedded tissue sections were deparaffined and rehydrated in an ethanol gradient. Sections were stained in Harris hematoxylin solution, differentiated in 1% acid alcohol and incubated in 0.2% ammonia water for color development. Counterstaining was performed in eosin Y solution, followed by dehydration. Finally, sections were mounted using a xylene-based mounting medium, and images were acquired using a light microscope (Nikon, ECLIPSE Ni-E) fitted with a digital camera (Canon, EOS 650D DS126371).

### Oil-Red-O staining

Mice livers were isolated and embedded in OCT compound (VWR, 25608-930). After freezing at −20 °C, livers were sectioned using a cryotome (Leica, CM1950) at 5-μm thickness and mounted on glass slides, air-dried for 30 min and fixed in ice-cooled 4% formaldehyde for 10 min. Slides were rinsed thrice with distilled water, air-dried, and then immersed in absolute propyl alcohol at 60°C for 5 min. Slides were stained with pre-warmed 0.5% Oil-Red-O solution (Sigma-Aldrich, U.S.A.) at 60°C for 10 min. Thereafter, samples were differentiated in 85% propyl alcohol for 5 min and rinsed twice with distilled water. Nuclei were counterstained with Mayer’s hematoxylin (Sigma-Aldrich, U.S.A.) for 30 s. Slides were washed with running tap water for 3 min and mounted with aqueous mounting medium (DAKO, Denmark).

### Biochemical and immunological assays

The serum cholesterol and triglyceride levels were quantified with commercial kits (Biosino Bio-technology, China) according to the manufacturer’s instructions. Alanine aminotransferase (ALT) and aspartate aminotransferase (AST) were assayed using an ADVIA 2400 Chemistry System analyzer (Siemens, Tarrytown, NY, U.S.A.) at the department of Laboratory Medicine, the First Affiliated Hospital of Xi’an Jiaotong University. Triglyceride and total cholesterol levels were assayed in liver samples using GPO-PAP method by commercial kits (Wako, Osaka, Japan) according to the manufacturer’s instructions. In brief, tissues were immersed in saline (tissue weight [g]: saline [ml] = 1:9), homogenized on ice and centrifuged at 2500 rpm for 10 min. Supernatant was collected for assay of protein content by the BCA method. Samples were added according to the manufacturer’s instructions. Detection was performed on a Microplate Reader. TG or TC content = [(sample OD value - Blank OD value) / (calibration OD value - Blank OD value)] × calibrator concentration ÷ sample protein concentration.

### Statistical analysis

Human samples distribution was determined by the Kolmogorov–Smirnov normality test and were analyzed by Wilcoxon signed-rank test or two-tailed, unpaired, Student’s *t*-test or Welch’s *t-*test. The chi-square test was used for categorical variables that are presented as numbers and percentages. A two-tailed, unpaired, Student’s *t*-test or one-way ANOVA were applied for the mouse experiments. Post-hoc comparison was performed using Bonferroni test. Data are reported as the mean ± SEM. Volcano plot was performed using R package “ggplot2” (https://CRAN.R-project.org/package=ggplot2). PCA analysis was performed using R package “ggfortify” (https://CRAN.R-project.org/package=ggfortify). Correlation network was performed using Cytoscape. The results were analyzed with GraphPad Prism 8.0 (GraphPad Software Inc, San Diego, CA, U.S.A.) or R software (version 3.6.1). *P*<0.05 was considered significant.

## Results

### GUDCA is reduced in glucose metabolic disorders

**Figure 1:**
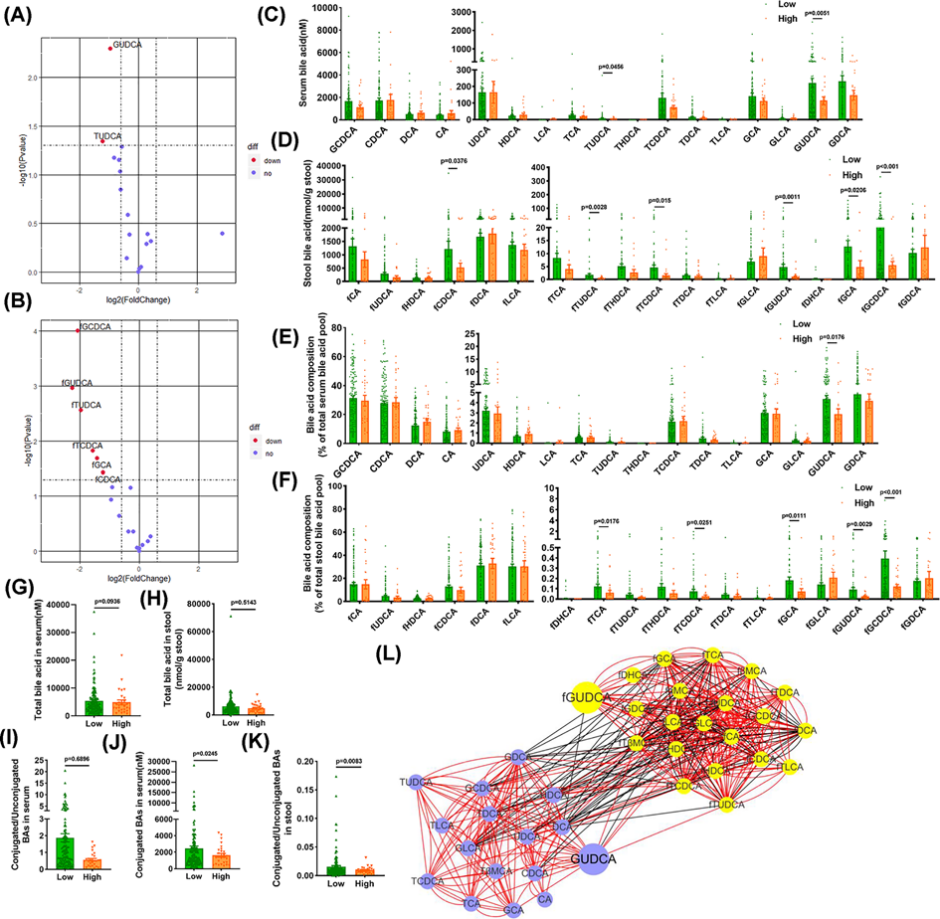
Changes of bile acid metabolism in patients with glucose metabolic disorders Collected patients in our metabolic cohort, stool and serum samples were obtained. (**A**) Volcano plot of bile acids in the serum (High vs. Low group). (**B**) Volcano plot of bile acids in the stool (High vs. Low group). (**C**) Levels of bile acids in the serum samples. (**D**) Levels of bile acids in the stool samples. (**E**) Composition of bile acids in serum. (**F**) Composition of bile acids in stool. (**G**) Total bile acid levels in serum samples. (**H**) Total bile acid levels in stool samples. (**I**) Ratios of conjugated to unconjugated bile acids in serum. (**J**) The level of conjugated bile acids in serum. (**K**) Ratios of conjugated to unconjugated bile acids in stool. (**L**) Correlation network of bile acids in the serum and feces. Purple means serum bile acids, yellow means fecal bile acids. Using Spearman correlation coefficient, red edges means *r* > 0, black edges means *r* < 0, all presented edges’ *P* are under 0.05 (*n*=135 for Low group, *n*=28 for High group). Data are shown as the mean ± SEM and analyzed by Welch’s *t* test.

To explore the changes of the BA metabolite profile in patients with hyperglycemia, a total of 163 serum and stool samples from the patients in our metabolic disease cohort were collected. The average age of this cohort was 59.8 ± 0.8 years. These samples were divided into two groups according to whether the HbA1c was greater than 6.5 (High group) or less than 6.5 (Low group). Baseline characteristics of the cohort were summarized in Table 1. High group patients have higher fasting glycaemia, lower HDL and have a trend of higher triglycerides and body mass index. Ultra-performance liquid chromatography-coupled time-of-flight mass spectrometry (UPLC-ESI-QTOFMS) metabolite profiling was applied to quantitate the levels of all BAs in the serum and stool samples. Volcano plots indicated that both GUDCA and TUDCA in the serum and stool samples were significantly lower in the High group than in the Low group (Figure 1A,B). Levels of BAs in the serum and stool samples are presented in Figure 1C,D, respectively. GUDCA and TUDCA were reduced in serum and stool of the High group as compared with the Low group, and the decline of GUDCA level was more striking than TUDCA. Analysis of the composition ratio of BAs showed that the proportion of GUDCA in the total BA was decreased in both serum and stool samples in the High group as compared with the Low group (Figure 1E,F).

The amount of total BAs was similar in serum and stool samples between the Low and High groups (Figure 1G,H). In serum, the ratios of conjugated to unconjugated BAs were not different significantly, while the amount of conjugated BAs was significantly lower in the High group compared with the Low group (Figure 1I,J). In addition, the ratios of conjugated to unconjugated BAs were reduced in stool samples in the High group as compared with the Low group (Figure 1K). No significant differences in the ratios of primary to secondary BAs and the ratios of 12α-OH to non-12α-OH BAs in serum and stool samples were observed (Supplementary Figure S1A–D). The gut bacteria can biotransform the primary BAs to secondary BAs and vice versa. A correlation network of BAs in the serum and feces of our metabolic disease cohort was generated using Cytoscape and indicated interconversion of BAs and connection of serum BAs to fecal BAs. GUDCA was the hub of maximal changes in serum and fecal BAs in metabolic disorders (Figure 1L).

### GUDCA inhibits ER stress-related genes in HFD-fed mouse liver

**Figure 2:**
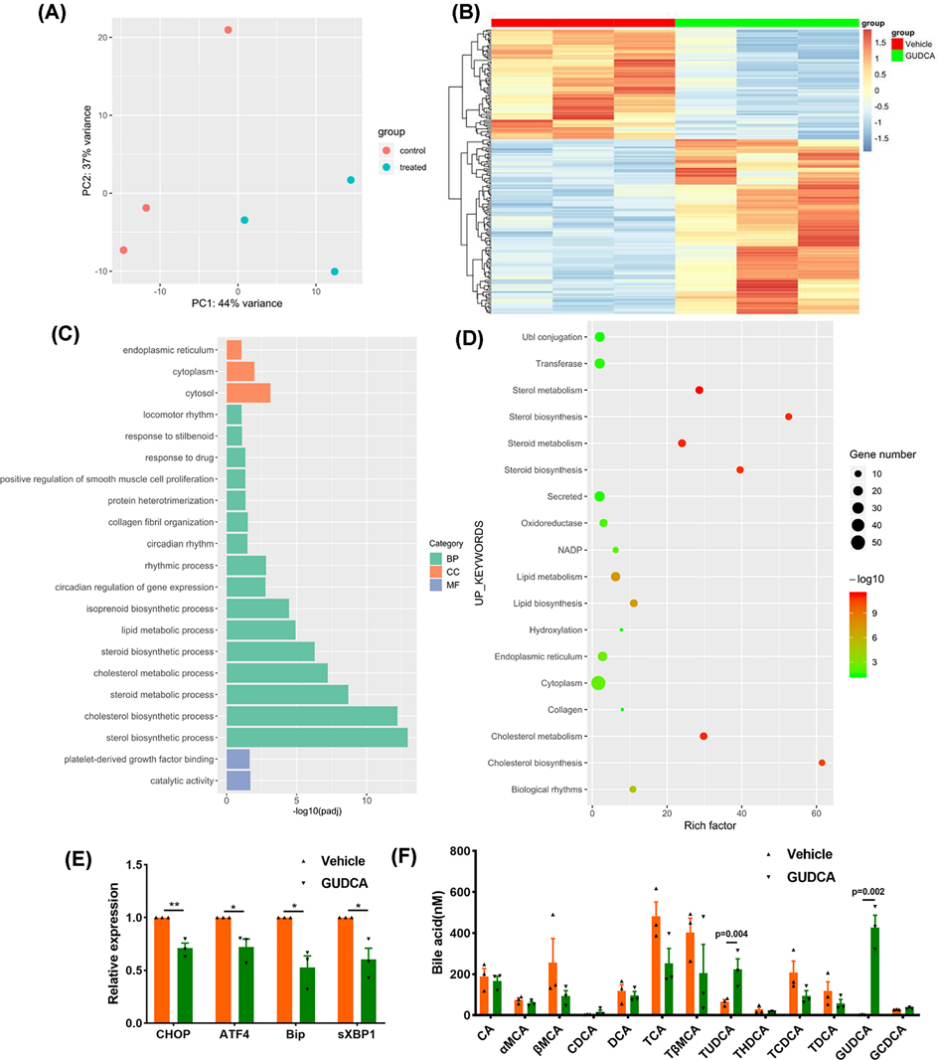
RNA-seq and real-time quantitative PCR results of vehicle or GUDCA-treated mice in liver HFD-fed mice were treated with vehicle or GUDCA (80mg/kg/d) for 1 week. (**A**) PCA for vehicle- and GUDCA-treated mice. (**B**) Heatmap of differential genes. (**C**) GO terms. (**D**) UP_KEYWORDS terms. (**E**) Quantitative real-time PCR measurement of CHOP, ATF4, Bip and sXBP1 mRNAs. (**F**) Bile acid levels in the serum after 1-week GUDCA treatment (*n* = 3/group); BP, biological process; CC, cellular component, MF, molecular function. Data are shown as the mean ± SEM and analyzed by two-tailed unpaired Student’s *t* test; *P<0.05, **P<0.01 versus control.

Next, we investigated the function of GUDCA in diet-induced metabolic disorders in mice. To explore the mechanism of GUDCA in regulating hepatic metabolism, RNA sequencing (RNA-seq) was analyzed to reveal the mRNA profiles in livers of HFD-fed mice with or without GUDCA administration. Principal component analysis (PCA) revealed that HFD-induced mRNA profile was substantially altered by GUDCA treatment (Figure 2A). Transcriptomic analysis revealed 116 up-regulated and 73 down-regulated genes in GUDCA+HFD-treated mice as compared with vehicle+HFD-treated mice (Figure 2B). Gene Ontology (GO) analysis showed that GUDCA differentially regulated 189 genes involved in biological processes including sterol, cholesterol and steroid biosynthesis, lipid metabolism, circadian rhythm and regulation, and the cellular component in cytosol, cytoplasm and endoplasmic reticulum, and molecular function including catalytic activity and platelet-derived growth factor binding (Figure 2C). UP_KEYWORDS analysis indicated that sterol, cholesterol, steroid biosynthesis and metabolism, cytoplasm and endoplasmic reticulum term were significantly altered by GUDCA (Figure 2D). The top 10 enriched Kyoto Encyclopedia of Genes and Genomes (KEGG) pathways are presented in Supplementary Figure S2A. The term of endoplasmic reticulum identified by GO and UP_KEYWORDS analyses led us to study the ER stress-related genes, which are known to involved in HFD-induced liver diseases. Real-time quantitative PCR was used to confirm the expression of ER stress-related genes. The results showed significant downregulation of the unfolded protein response (UPR) target genes including C/EBP homologous protein (CHOP), activating transcription factor4 (ATF4), binding immunoglobulin protein (Bip) and spliced X-box binding protein (sXBP1) in GUDCA+HFD-treated mice as compared with vehicle+HFD-fed mice (Figure 2E). The body weight, food-intake, blood lipids, ALT and AST levels of mice had no statistical differences between GUDCA+HFD-treated mice and vehicle+HFD-treated mice (Supplementary Figure S2B–I), suggesting that the decrease in ER stress-associated genes in GUDCA-treated mice was not attributed to metabolic improvements. Analysis of BA profiles revealed that GUDCA and TUDCA levels were increased in GUDCA-treated mice, whereas other BA metabolites were unaffected (Figure 2F). The analysis of total BA and the composition of BA profiles are displayed in Supplementary Figure S2J–M.

### GUDCA ameliorates ER stress induced by palmitate

**Figure 3:**
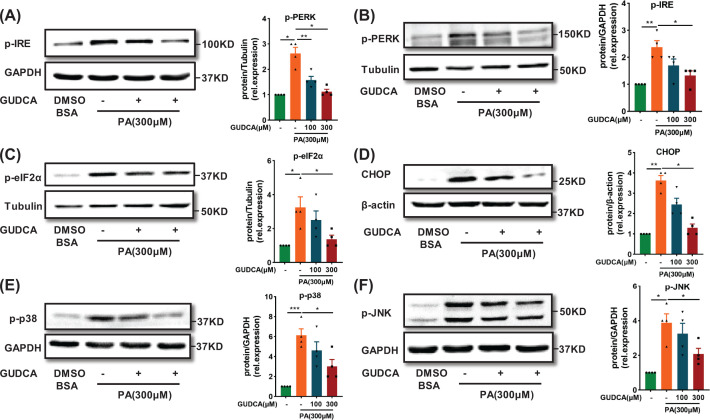
GUDCA reduces ER stress induced by palmitate *in vitro* (**A**) Representative western blot analysis of ER stress markers p-IRE (**B**) p-PERK (**C**) p-eIF2α (**D**) CHOP (**E**) p-p38 and (**F**) p-JNK in HepG2 that were treated with PA with or without GUDCA, for 24 h. GAPDH, Tubulin or β-actin was used as loading control (*n* = 4/group). All data are presented as the mean ± SEM and analyzed by one-way ANOVA followed by the Bonferroni post hoc test; *P<0.05, **P<0.01, ***P<0.001 versus control.

To confirm the role of GUDCA in HFD-induced hepatic ER stress, the effect of GUDCA on saturated fatty acid PA-induced ER stress in HepG2 cells was examined [26]. PA significantly enhanced the levels of ER stress markers in HepG2 cells, including phosphorylated-mammalian inositol requiring kinase (p-IRE), phosphorylated-eukaryotic translation initiation factor 2 subunit alpha (p-eIF2α) and CHOP (Supplementary Figure S3A–D). GUDCA co-treatment reduced PA-stimulated ER stress markers, indicating the protective effect of GUDCA against PA-induced ER stress (Figure 3A–D). Since the Jun N-terminal kinase (JNK) and p38 mitogen-activated protein kinases (p38 MAPK) signaling pathways control cellular adaptive responses to stresses [27], changes of phospho-JNK(p-JNK) and phospho-p38 MAPK (p-p38) were further examined. Western blotting results showed that GUDCA attenuated PA-induced increase of p-p38 and p-JNK in HepG2 cells (Figure 3E,F). Taken together, these data demonstrated that GUDCA treatment ameliorated PA-induced ER stress in HepG2 cells.

### GUDCA blocks palmitate-induced apoptosis

**Figure 4:**
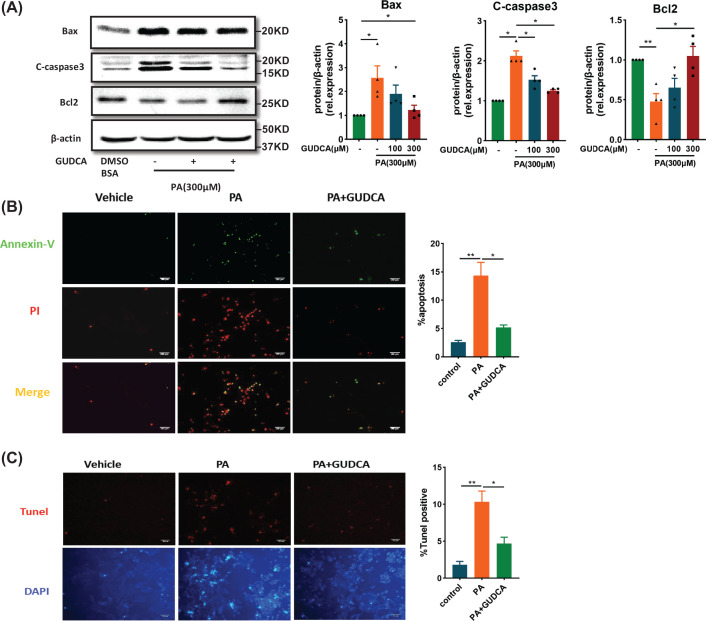
GUDCA decreases PA-induced apoptosis *in vitro* (**A**) Representative Western blot analysis of apoptosis-related protein C-caspase3, Bax and Bcl2 in HepG2 treated with PA with or without GUDCA for 24 h. β-Actin was used as loading control (*n* = 4/group). (**B**) Annexin V-FITC/PI double staining for apoptotic HepG2 treated with BSA, PA (300 μM), or PA (300 μM) + GUDCA (300 μM) for 24 h. Early apoptotic cells were marked only in green, whereas late apoptotic cells were presented in red with green (*n* = 3/group). (**C**) TUNEL assay for apoptotic HepG2 that were treated BSA, PA, or PA + GUDCA for 24 h. All apoptotic cells were marked in red (*n* = 3/group); scale bars: 100 µm. All data are presented as the mean ± SEM and analyzed by one-way ANOVA followed by the Bonferroni post hoc test; *P<0.05, **P<0.01 versus control.

Endoplasmic reticulum stress is one of the major mechanisms leading to apoptosis through prolonged activation of IRE or induction of CHOP [28]. Therefore, the effect of GUDCA on PA-induced apoptosis in HepG2 cells was investigated. GUDCA co-treatment reduced PA-induced increase of pro-apoptotic Bax and Cleaved caspase 3 (C-caspase3) and decrease of the anti-apoptotic Bcl2 (Figure 4A). In parallel, Annexin-V/PI and TUNEL staining showed that PA treatment significantly increased Annexin-V/PI and TUNEL-positive cells as compared with the control group, which was reversed by GUDCA treatment (Figure 4B,C). Collectively, these data indicated that GUDCA was capable of decreasing PA-induced apoptosis in HepG2 cells.

### GUDCA stabilizes intracellular calcium homeostasis in palmitate-induced cells

**Figure 5:**
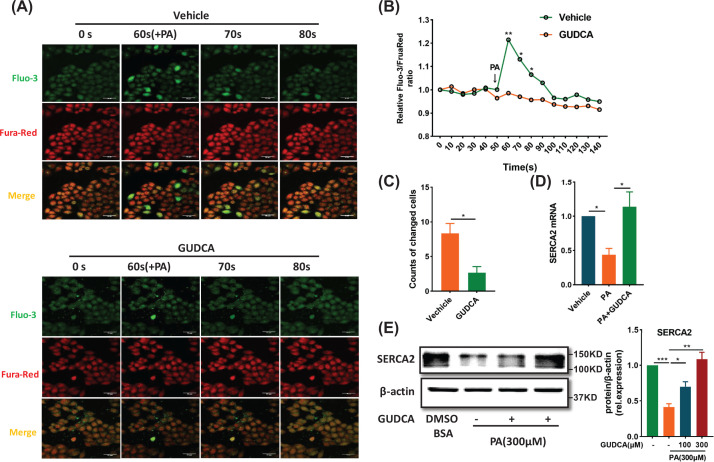
Effect of GUDCA in stabilizing calcium homeostasis *in vitro* (**A**) Comparisons of fluorescence intensity in response to PA between vehicle and GUDCA-pretreated cells. The cells were analyzed using Fluo-3/Fura-Red dual-labeling confocal microscopy; scale bars: 50 µm (*n* = 3/group). (**B**) Relative Fluo-3/FruaRed ratio and (**C**) the number of cells with significant changes in fluorescence. (**D**) Quantitative real-time PCR and (**E**) Immunoblot measurement of SERCA2 in the HepG2 with indicated interventions (*n* = 4/group). All data are presented as the mean ± SEM and analyzed by two-tailed unpaired Student’s *t* test for (A), one-way ANOVA followed by the Bonferroni post hoc test for (B) and (C); *P<0.05, **P<0.01, ***P<0.001 versus control.

Endoplasmic reticulum provides a platform for the crosstalk among many cellular signaling pathways and metabolic regulatory events through calcium fluxes [29]. To examine the role of GUDCA in calcium flux, we monitored the changes in cytoplasmic calcium concentration using Fluo-3/Fura Red fluorescence ratios [30]. No differences in baseline cytoplasmic calcium concentrations between vehicle and GUDCA-pretreated cells were observed. PA treatment caused calcium efflux and GUDCA pretreatment reduced PA-stimulated calcium efflux (Figure 5A–C). Since sarcoplasmic/ER Ca^2+^ ATPase (SERCA) is principally responsible for maintaining calcium homeostasis in ER [31], the GUDCA effect on SERCA2 expression was assessed. The PA-reduced SERCA2 expression was restored by GUDCA at both the mRNA and protein levels (Figure 5D,E). These data indicated that GUDCA maintained intracellular calcium homeostasis to prevent PA-induced ER stress in HepG2 cells.

### GUDCA treatment improves insulin sensitivity in HFD-fed mice

**Figure 6:**
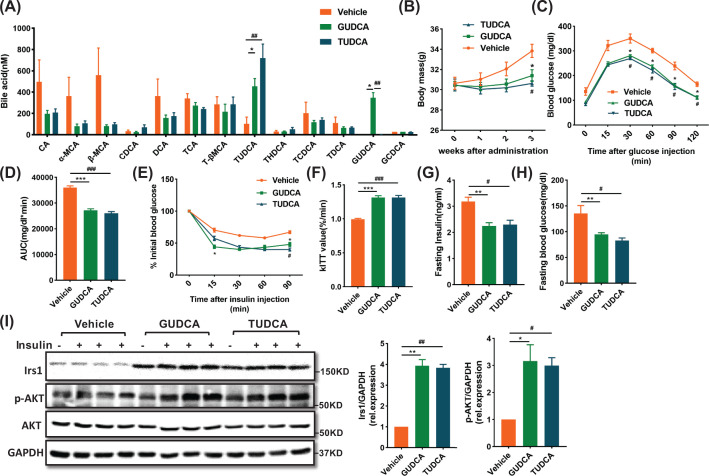
GUDCA supplementation blocks diet-induced insulin resistance HFD-fed mice were treated with vehicle or GUDCA (80 mg/kg/d) or TUDCA (350 mg/kg/d) during the last 3 weeks (*n* = 5–8/group). (**A**) Bile acid profiles in the serum after 3 weeks GUDCA or TUDCA treatment. (**B**) Body weight changes after administration. (**C**) GTT, (**D**) AUC, (**E**) ITT, (**F**) kITT value, (**G**) fasting insulin levels and (**H**) fasting glucose levels. (**I**) Irs1 and Akt signaling in liver. Data are presented as mean ± SEM and analyzed by one-way ANOVA followed by the Bonferroni post hoc test; *P<0.05, **P<0.01, ***P<0.001 GUDCA versus vehicle; ^#^*P*<0.05, ^##^*P*<0.01, ^###^*P*<0.01 TUDCA versus vehicle.

To further verify the role of GUDCA in alleviating diet-induced metabolic dysfunction and ER stress *in vivo*, we fed mice with HFD for 8 weeks to induce metabolic dysfunction and administered mice with either vehicle, GUDCA or TUDCA during the last 3 weeks. TUDCA was used as a positive control since it was an established ER stress inhibitor capable of improving metabolic dysfunction [22]. Analysis of BA profiles indicated that GUDCA or TUDCA levels were markedly elevated in GUDCA or TUDCA treated mice, whereas other BA metabolites levels remained unchanged (Figure 6A). Detailed analysis of BA composition profile is provided in Supplementary Figure S4A–D. There were no differences in the total content of BAs due to either treatment (Supplementary Figure S4E). Moreover, the expression of BA efflux pump, ATP-binding cassette subfamily C member 3 and 11 (Abcc3 and Abcc11), and the BA synthetic enzyme, cholesterol 7α hydroxylase (Cyp7a1) were unchanged in GUDCA- and TUDCA-treated mice as compared with vehicle-treated in liver (Supplementary Figure S4F–4H). Compared with vehicle treatment, both GUDCA and TUDCA treatments substantially reduced weight gain (Figure 6B). Glucose and insulin tolerance tests were performed to investigate the role of GUDCA in HFD-related glucose homeostasis dysfunction. GTT revealed that GUDCA and TUDCA treatments significantly restored glucose tolerance as compared with vehicle-treated mice (Figure 6C,D). ITT and constant for insulin tolerance test (kITT) values demonstrated that insulin sensitivity was substantially increased after GUDCA and TUDCA treatments (Figure 6E,F). Fasting insulin and blood glucose levels of GUDCA- and TUDCA-treated mice were lower than those of vehicle-treated mice (Figure 6G,H). The effects of GUDCA and TUDCA on insulin signaling in liver tissue were then examined. Both GUDCA and TUDCA abolished the HFD-mediated reduction of insulin receptor substrate1(Irs1) and phosphorylated protein kinase B (p-Akt) (Ser473) in liver as compared with vehicle treatment (Figure 6I). These results suggested that GUDCA prevented HFD-induced insulin resistance in mice with an efficacy equivalent to TUDCA.

### GUDCA treatment protects mice from HFD-induced hepatic steatosis

**Figure 7:**
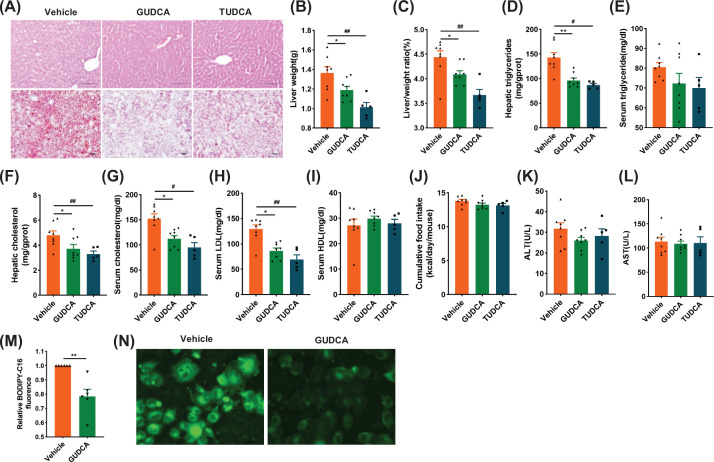
Administration of GUDCA reverses HFD-induced hepatic steatosis (**A**) Representative H&E staining (upper) and Oil Red O staining (lower) of liver sections (*n* = 5–8/group, three images/mouse); scale bars: 100 µm. (**B**) Liver weights. (**C**) Liver weight-to-body weight ratios. (**D**) Liver and (**E**) serum triglyceride content. (**F**) Liver and (**G**) serum cholesterol content. (**H**) Serum LDL and (**I**) HDL. (**J**) Cumulative food intake. (**K**) Serum ALT and (**L**) AST levels. (**M**) The ability of fatty acid uptake in HepG2 cells pre-treated with GUDCA or vehicle for 24 h was measured fluorometrically by using BODIPY-C16. (**N**) Fluorescence images of BODIPY -C16 (green) were acquired (400×; *n* = 5–8/group). All data are presented as the mean ± SEM and analyzed by one-way ANOVA followed by the Bonferroni post hoc test. *P<0.05, **P<0.01 GUDCA versus vehicle; ^#^*P*<0.05, ^##^*P*<0.01 TUDCA versus vehicle.

Since BAs are produced from liver cholesterol catabolism and the strong association between T2DM and MAFLD, the effect of GUDCA on ectopic lipid accumulation in liver was studied. Both H&E and Oil Red O staining showed a reduction in hepatic lipid accumulation in mice treated with either GUDCA or TUDCA as compared with vehicle-treated mice (Figure 7A). Both GUDCA and TUDCA reduced liver weights and liver/body weight ratios as compared with those of the control group (Figure 7B,C). Epididymal fat mass and adipocyte size were reduced by GUDCA and TUDCA treatment as compared with vehicle-treated mice (Supplementary Figure S4I and J). The levels of hepatic triglycerides, hepatic and serum cholesterol, and serum low density lipoprotein (LDL) were lower in GUDCA and TUDCA-treated mice, while no significant differences were found in serum triglyceride and high-density lipoprotein (HDL) concentrations and food-intake as compared with vehicle-treated mice (Figure 7D–J). Of note, GUDCA did not change serum ALT and AST levels, indicating GUDCA was not hepatotoxic (Figure 7K,L). In addition, we employed BODIPY-C16, a fatty acid analogue with green fluorescence that has been used to measure the efficiency of fatty acid uptake in many cell types [32], to evaluate the effect of GUDCA on fatty acid uptake. The results showed the decrease of fatty acid uptake in HepG2 cells pretreated with GUDCA as compared with control cells (Figure 7M,N). These results showed that GUDCA protected mice from HFD-induced hepatic steatosis.

### GUDCA inhibits ER stress-related pathway and apoptosis in the liver of HFD-fed mice

**Figure 8:**
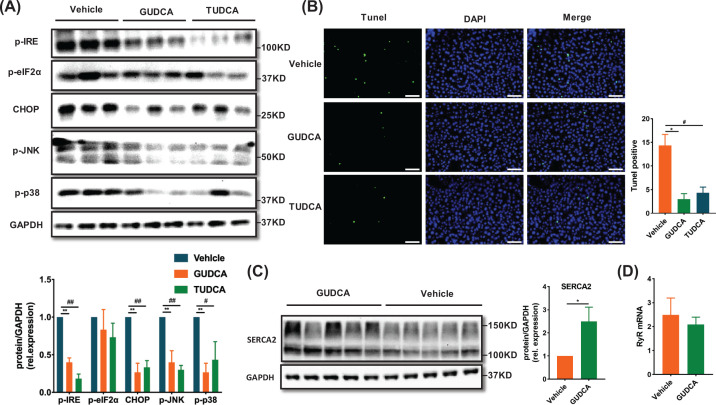
GUDCA attenuates HFD-induced ER stress activation and apoptosis in liver (**A**) Western blot analysis of p-IRE, p-eIF2α, CHOP, p-JNK and p-p38 protein expression in livers and the statistical graph (*n* = 3/group). (**B**) Representative staining of the livers with TUNEL in mice; scale bars: 100 µm. (**C**) Representative immunoblot of SERCA2. (**D**) Quantitative real-time PCR measurement of RyR in mice (*n* = 5-8/group). All data are presented as the mean ± SEM and analyzed by one-way ANOVA followed by the Bonferroni post hoc test for (A) and (B), two-tailed unpaired Student’s *t* test for (C) and (D). *P<0.05, **P<0.01 GUDCA versus vehicle; ^#^*P*<0.05, ^##^*P*<0.01 TUDCA versus vehicle.

Finally, the expression of UPR-related proteins and apoptosis in liver tissues of mice treated by GUDCA or TUDCA were measured. Generally, protein levels of p-IRE, and CHOP, p-p38 and p-JNK were reduced, along with a trend toward reduced p-eIF2α expression in GUDCA and TUDCA-treated mice as compared with vehicle-treated (Figure 8A). In addition, TUNEL staining positive cells were also significantly reduced in mouse livers after being treated with GUDCA and TUDCA (Figure 8B). The expression of SERCA2 was increased in GUDCA-treated mice as compared with vehicle-treated, while ryanodine receptor (RyR) expression was unchanged (Figure 8C,D). Taken together, these findings suggested that GUDCA alleviated diet-related metabolic disorders associated with inhibiting ER stress and apoptosis in liver.

## Discussion

Changes in energy balance in mice fed a high-fat diet is associated with changes in the gut microbiota and increased BA metabolism [33]. The α-glucosidase inhibitor miglitol affects BA metabolism and ameliorates obesity and insulin resistance in diabetic mice [34]. BA composition regulates GPR119-dependent intestinal lipid sensing and food intake regulation [35]. These results indicate that BAs are indicators and regulators of metabolic disorders. To elucidate the role and mechanism of BA in modulating metabolic disorders, it is of great interest to identify changes in BA composition, find the key endogenous BA metabolites and determine its metabolic target(s). In the present study, GUDCA was significantly reduced in both serum and stool in patients with glucose metabolic disorders. Supplementation of GUDCA effectively alleviated insulin resistance and hepatic steatosis in HFD-induced mice with inhibition of ER stress. These findings indicate that GUDCA might be a promising therapeutic agent for diet-induced metabolic disorders.

The emerging importance of the intestinal microbiota in host health and pathogenesis of several metabolic disorders, including obesity, T2DM and MAFLD has been established [9–10]. The host-gut microbiota interaction is central to BA metabolism and signaling and is essential for the maintenance of metabolic health [18]. In the present study, we found that both GUDCA and TUDCA were significantly decreased in serum and feces in the High group, and the change of GUDCA was more significant than TUDCA. Bile salt hydrolases (BSH) is produced by the gut microbiota, regulating hydrolysis of conjugated BA. BSH is active in Lactobacillus, Firmicutes, Enterococcus, Clostridium and Bacteroides, and deconjugates conjugated BAs [36]. Metformin increases the levels of conjugated BAs by reducing the abundance of B. fragilis and its BSH activity to alleviate T2DM [17]. The possible mechanism in the present study is that the abundance of BSH-producing bacteria is reduced, which deconjugates BAs, reduces the concentration of GUDCA and TUDCA, thereby exacerbating hyperglycemia.

It is generally accepted that a surplus of nutrients and energy stimulates the biosynthetic pathways, which may lead to lipid and protein overloading in the ER. Several stimuli, including disturbances to the metabolic and calcium homeostasis, inflammation or oxidative stress [37,38], can trigger ER stress. To ameliorate ER stress and to ensure correct protein folding occurs in response to noxious stimuli, an adaptive signaling pathway known as the UPR, is initially triggered to restore ER proteostasis. If the UPR fails and ER homeostasis cannot be restored, cell death is activated [39]. Lipids accumulation is toxic for the hepatocyte and is pivotal for ER stress to cause insulin resistance and hepatic steatosis [40]. ER stress can disrupt insulin action through the activation of the arm of the UPR. IRE1α’s kinase function phosphorylates JNK kinase, inhibiting insulin signaling at the level of insulin receptor substrates [41]. Similarly, the PERK arm contributes to insulin resistance [42]. In addition, the UPR regulates hepatic lipid homeostasis. IRE1α-XBP1 arm was shown to be a crucial player in hepatic lipid metabolism through regulation of VLDL secretion and lipogenesis [43]. CD154, as a ligand of CD40 and key mediator of inflammation, may play a protective role in hepatic steatosis through a possible connection to PERK-eIF2α-CHOP arm [44]. The present study indicated that ER stress-related genes, mainly including IRE1 and PERK arm were significantly reduced in GUDCA-treated mice compared with vehicle-treated mice, and GUDCA ameliorated diet-induced metabolic disorders. It is worth noting that GUDCA decreased ER stress when lipid levels and body weight were not altered at 1 week. The results revealed that the consistent decrease of ER stress was due to an effect of GUDCA treatment, but not derived from the subsequent metabolic improvements. ER stress increases Abcc3, reduces Cyp7a1 and hepatic BAs content [45]. It's possible that a metabolic crosstalk exists between BAs and ER stress. However, total BAs, and Abcc3, Abcc11, Cyp7a1 mRNA levels were unchanged in this study, suggesting that there might be a dynamic equilibrium of total BAs in GUDCA-alleviated diet-induced metabolic disorders. In addition, the diet-induced metabolic disorders model used here was more complex and comprehensive than the tunicamycin-induced ER stress model.

Maintenance of calcium homoeostasis is of crucial importance for the proper functioning of cells. Calcium dyshomeostasis is associated with many pathological conditions, including metabolic disorders, neurological and heart diseases [29,46]. Abnormal changes in calcium equilibrium can lead to disrupted protein folding and cause accumulation of misfolded proteins in the ER. SERCAs, which are pumps that transport free Ca^2+^ from the cytosol to the ER lumen in an ATP-requiring manner. Pioglitazone alleviates high glucose and apoptosis by increasing SERCA2b transcription [47]. Disturbances in hepatic lipid composition during models of obesity in mice can inhibit the SERCA2, which induces ER stress and disrupts glucose homeostasis in the liver [31]. GUDCA treatment partially attenuated down-regulation in SERCA2 induced by HFD, mitigating Ca^2+^ depletion from the ER lumen and inhibition of ER stress, then alleviating metabolic diseases, which may be the mechanism throughout the present study.

In fact, age is a key factor for liver function, BA production and the crosstalk between BAs and metabolic functions [48]. The present study was carried out with patients approximately 60 years old and represented BA metabolism in elderly patients. Further studies should be performed in younger patients. Moreover, compared with the Low Group, the small number of patients in the High Group is a limitation of our study. The large difference in sample size between the two groups will reduce statistical efficiency, but it will not affect the correctness of statistical inference. Because *P* value calculations are based on at least the two-dimensional information of “statistics” and “degrees of freedom”, both of them take into account the sample size of the two groups.

It is noteworthy that the additional supplement of GUDCA increased the levels of GUDCA and TUDCA, but TUDCA treatment did not increase the relative concentration of GUDCA in the present study. This raises the possibility that the effect of GUDCA on metabolic disorders is related to the increase of TUDCA. The network of BAs transformation and interaction is highly complicated *in vivo*. The selection of taurine or glycine for BA conjugation is mainly controlled by the bile acid-CoA amino acid N-acyltransferase (BAAT) enzyme the BAAT enzyme is particularly inefficient at using glycine for conjugating bile acids in mice [49,50]. Moreover, the BSH produced by gut microbiome controls the hydrolysis of conjugated BA and involves in the GUDCA transformation to TUDCA. The specific mechanism underlying GUDCA-mediated up-regulation of TUDCA is worthy of further study. Since TUDCA acts as an antidiabetic modality with potential application in the treatment of T2DM [22], and a relatively small dose of GUDCA increased both GUDCA and TUDCA in present study, GUDCA might have great potential in clinical use.

## Conclusion

In conclusion, our study provides the first evidence that GUDCA is decreased in patients with glucose metabolic disorders. Further, we found that GUDCA exerts protective functions against diet-induced insulin resistance and hepatic steatosis with inhibiting ER stress, might promise a new treatment option for metabolic disorders in patients.

## Clinical perspectives

- We explored the alteration of bile acid metabolism profiling in individuals with hyperglycemia and identified GUDCA was reduced dramatically.
- Our study provided the evidence that GUDCA inhibited ER stress and apoptosis both *in vitro* and *in vivo*.
- We suggested that small-molecule agents, GUDCA might have therapeutic potential for the treatment of diet-induced insulin resistance and hepatic steatosis.

## Supplementary Material

Supplementary Figures S1-S5 and Table S1

## Data Availability

The complete RNA-Seq dataset is available from the GEO repository (GSE168822).

## Abbreviations

ALT: alanine aminotransferase
AST: aspartate aminotransferase
BA: bile acid
BAAT: bile acid-CoA amino acid N-acyltransferase
BSH: bile salt hydrolases
DCA: deoxycholic acid
DHCA: dehydrocholic acid
ER: endoplasmic reticulum
FGF19: fibroblast growth factor 19
FXR: farnesoid X receptor
GTT: glucose tolerance test
GUDCA: glycoursodeoxycholic acid
HbA1c: glycated hemoglobin A1c
HDL: high-density lipoprotein
HFD: high-fat diet
IR: insulin resistance
IRS1: insulin receptor substrate1
ITT: insulin tolerance test
LCA: lithocholic acid
LDL: low-density lipoprotein
MAFLD: metabolic associated fatty liver disease
NAFLD: non-alcoholic fatty liver disease
p-Akt: phosphorylated protein kinase B
T2DM: type 2 diabetes mellitus
TCA: taurocholic acid
TCDCA: taurochenodeoxycholic acid
TDCA: taurodeoxycholic acid
THDCA: taurohyodeoxycholic acid
TUDCA: tauroursodeoxycholic acid
UDCA: ursodeoxycholic acid
UPR: unfolded protein response
