# Supplementary material for: Glycoursodeoxycholic acid ameliorates diet-induced metabolic disorders with inhibiting endoplasmic reticulum stress
Source: Clin Sci (Lond). 2021 Jul 20;135(14):1689–706. doi: 10.1042/CS20210198 (PMC8302808; doi:10.1042/CS20210198)
Supplement: Supplementary Figures S1-S5 and Table S1 [file CS-2021-0198_supp.pdf]

Supplementary Figure 1

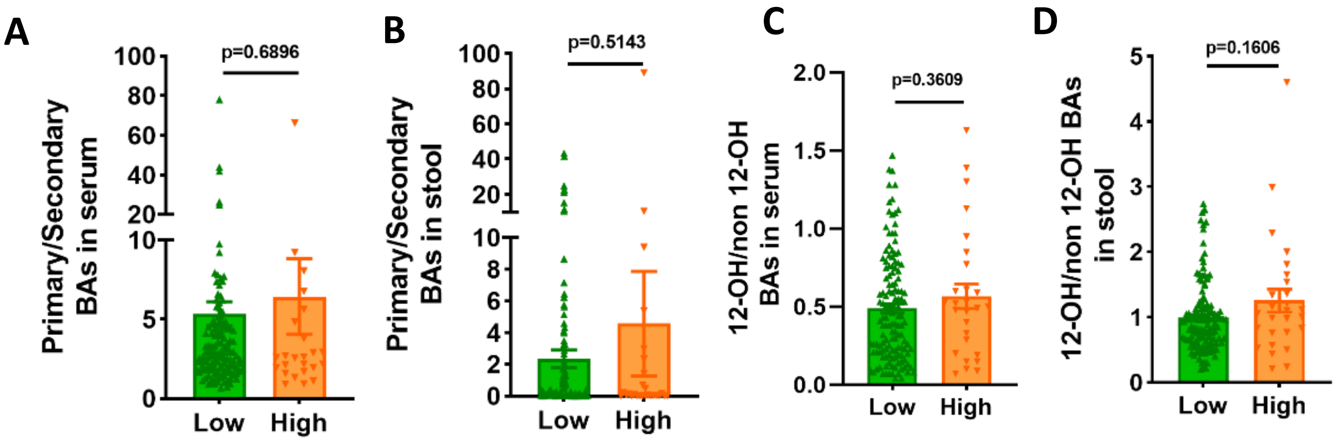

Supplementary Figure1. Levels and composition of bile acids in samples. A, Ratios of primary to secondary bile acids in the serum and **B,** stool. **C,** The ratios of 12α-OH to non 12α-OH bile acids in serum and **D,** stool. n =135 for Low group, n=28 for High group. Data are shown as the mean ± SEM and analyzed by Wilcoxon signed-rank test.

Supplementary Figure 2

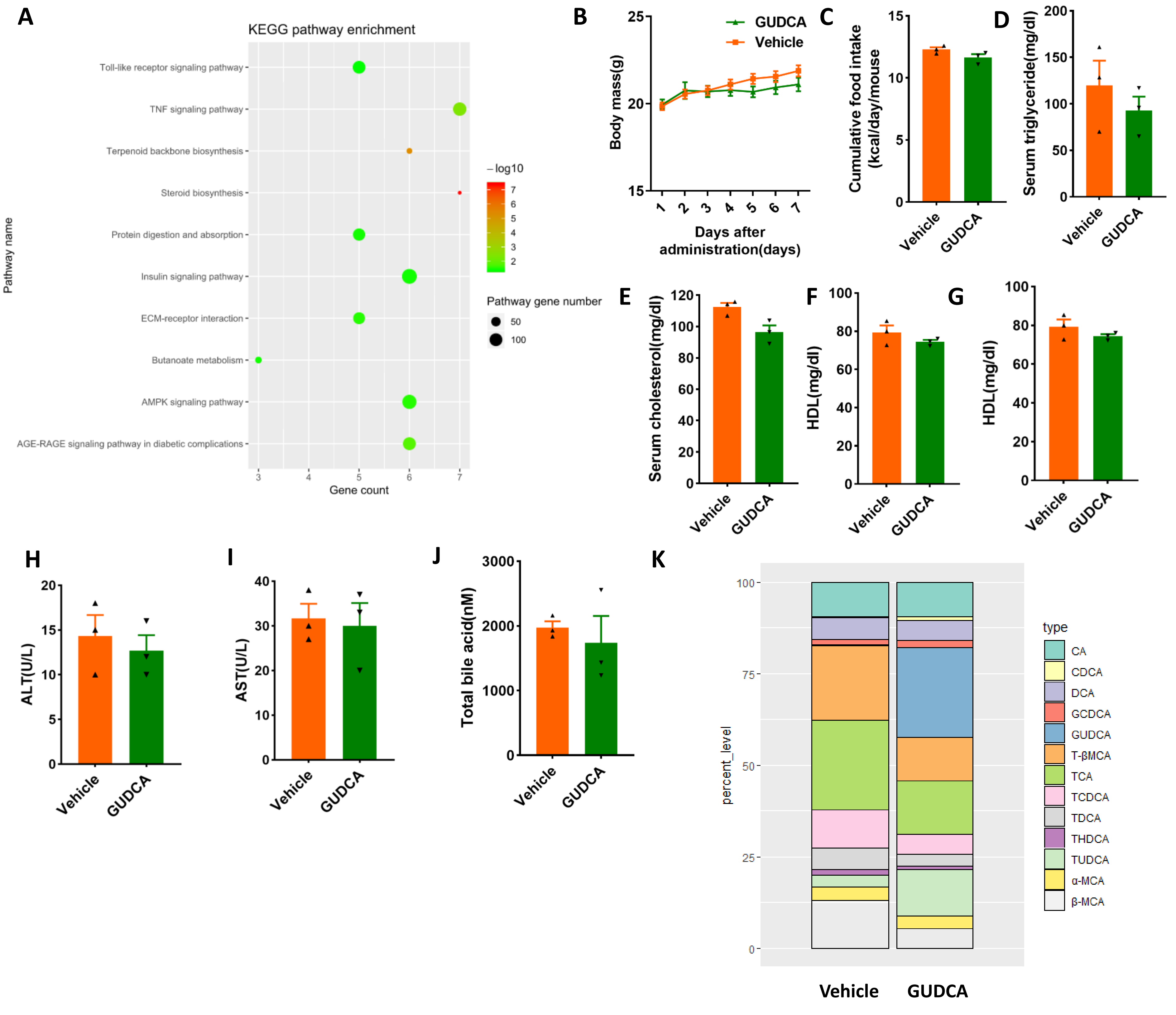

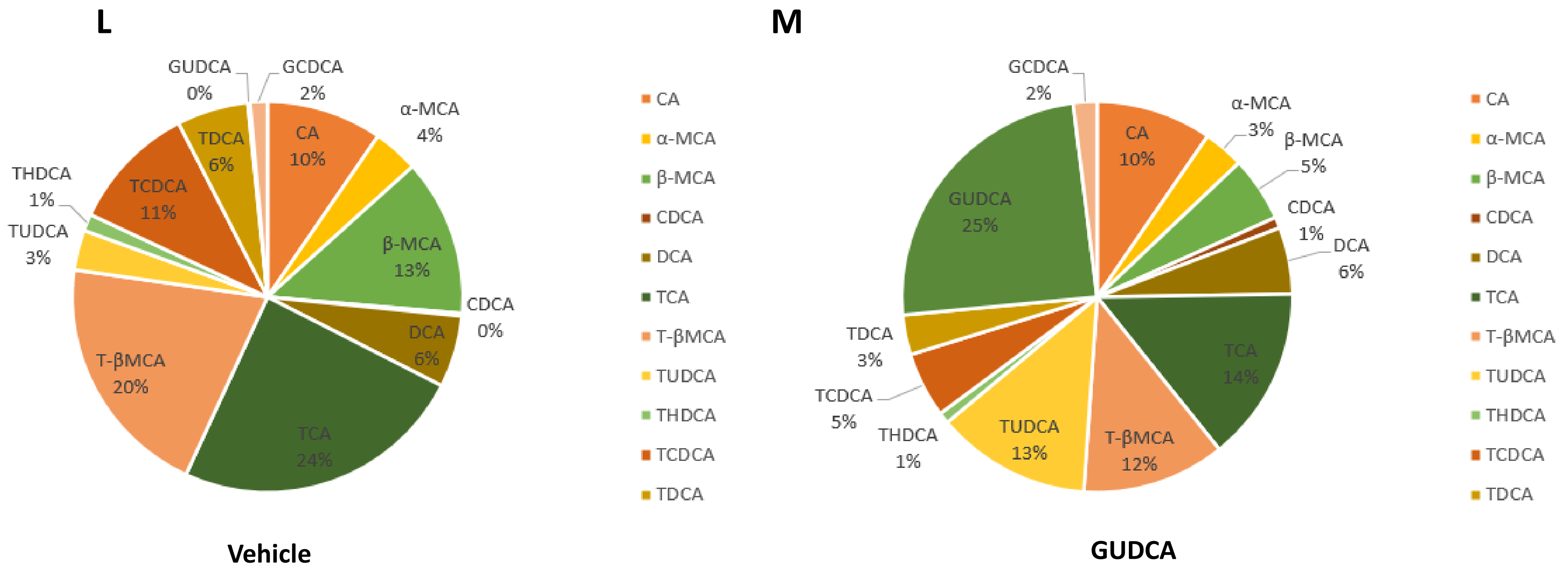

Supplementary Figure2. RNA-seq and biochemical indicators of mice. A, KEGG pathways. **B,** Body weight. **C,** Cumulative food intake. **D,** Serum triglyceride content. **E,** Serum cholesterol content. **F,** Serum LDL levels. **G,** Serum HDL levels. **H,** Serum ALT and **I,** AST levels. **J,** Total bile acids in serum. **K-M,** Composition of bile acids in serum. (n = 3/group). Data are shown as the mean ± SEM and analyzed by Two-tailed unpaired Student's t test.

Supplementary Figure 3

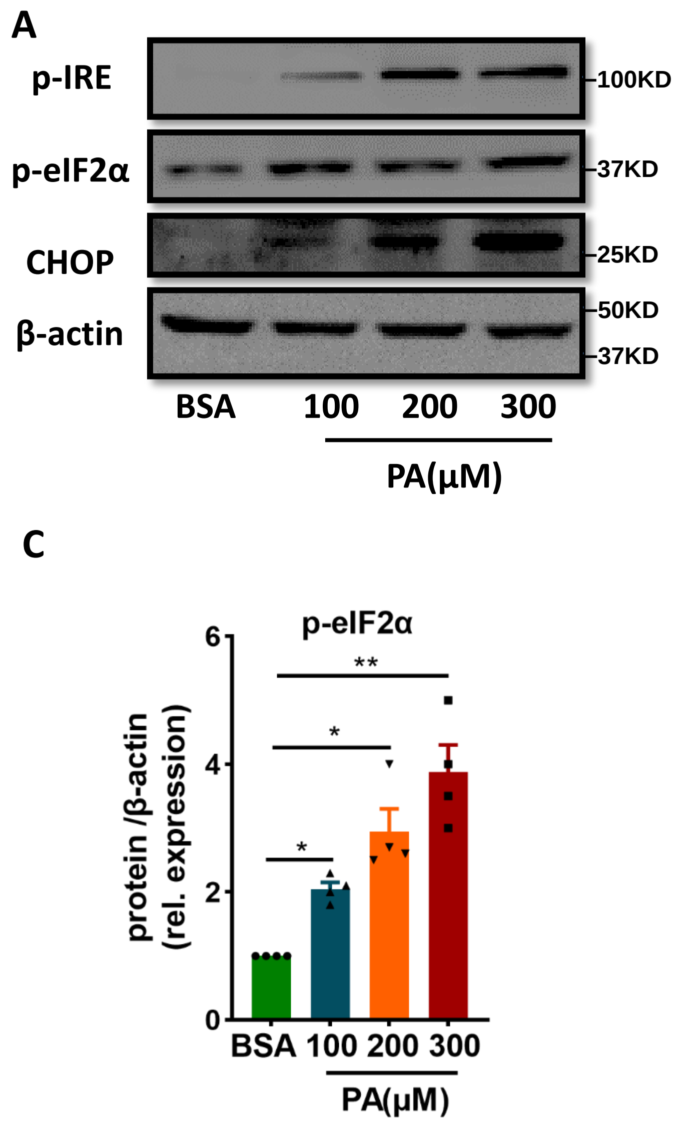

Supplementary Figure 3. Palmitate induces ER stress expression in dose dependent manners. A, p-IRE, p-eIF2α and CHOP expression were measured by western blot. **B-D,** The statistical graph (n=4/group). All data are presented as the mean ± SEM and analyzed by one-way ANOVA followed by the Bonferroni post hoc test. *$P < 0.05$, **$P < 0.01$ vs. control.

Supplementary Figure 4

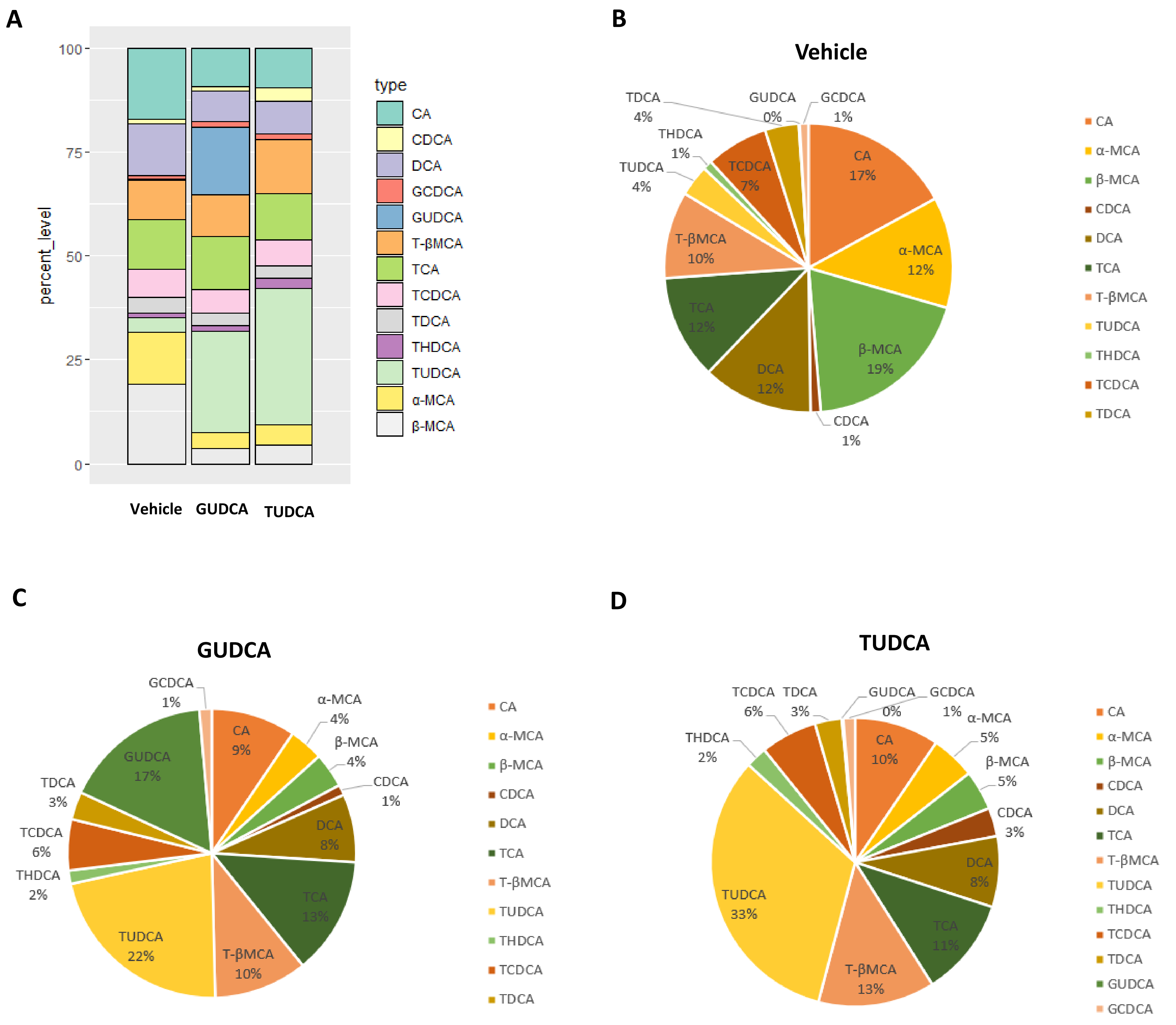

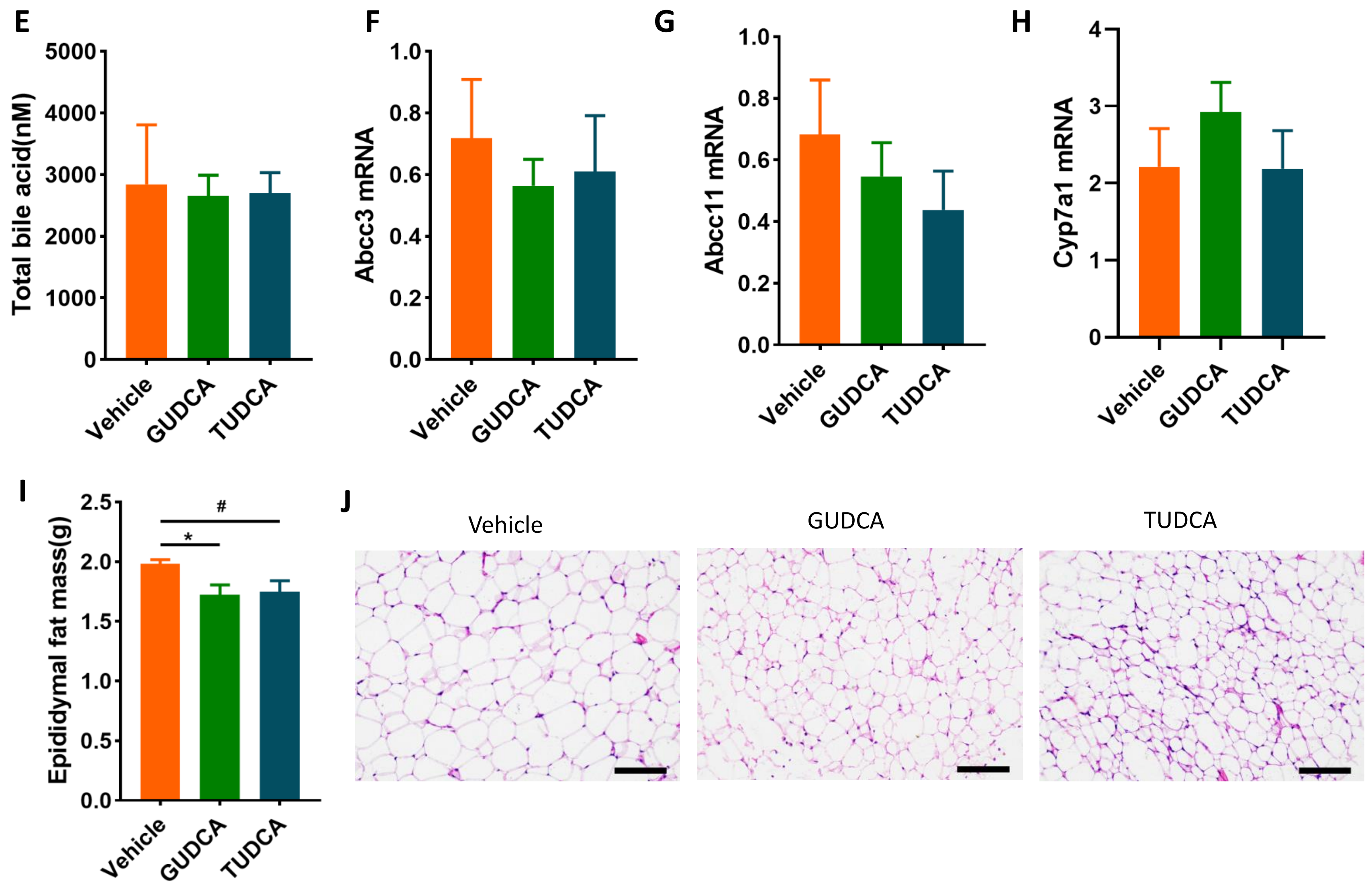

Supplementary Figure 4. A-D, Composition of bile acids in serum of vehicle, GUDCA or TUDCA treated mice. **E,** Total bile acid in serum. **F,** The mRNA expression of Abcc3 **G,** Abcc11 and **H,** Cyp7a1. **I,** Epididymal fat mass. **J,** H&E staining of inguinal adipose tissue (n = 5-8/group). Data are presented as the mean ± SEM and analyzed by one-way ANOVA followed by the Bonferroni post hoc test.

Supplementary Figure 5

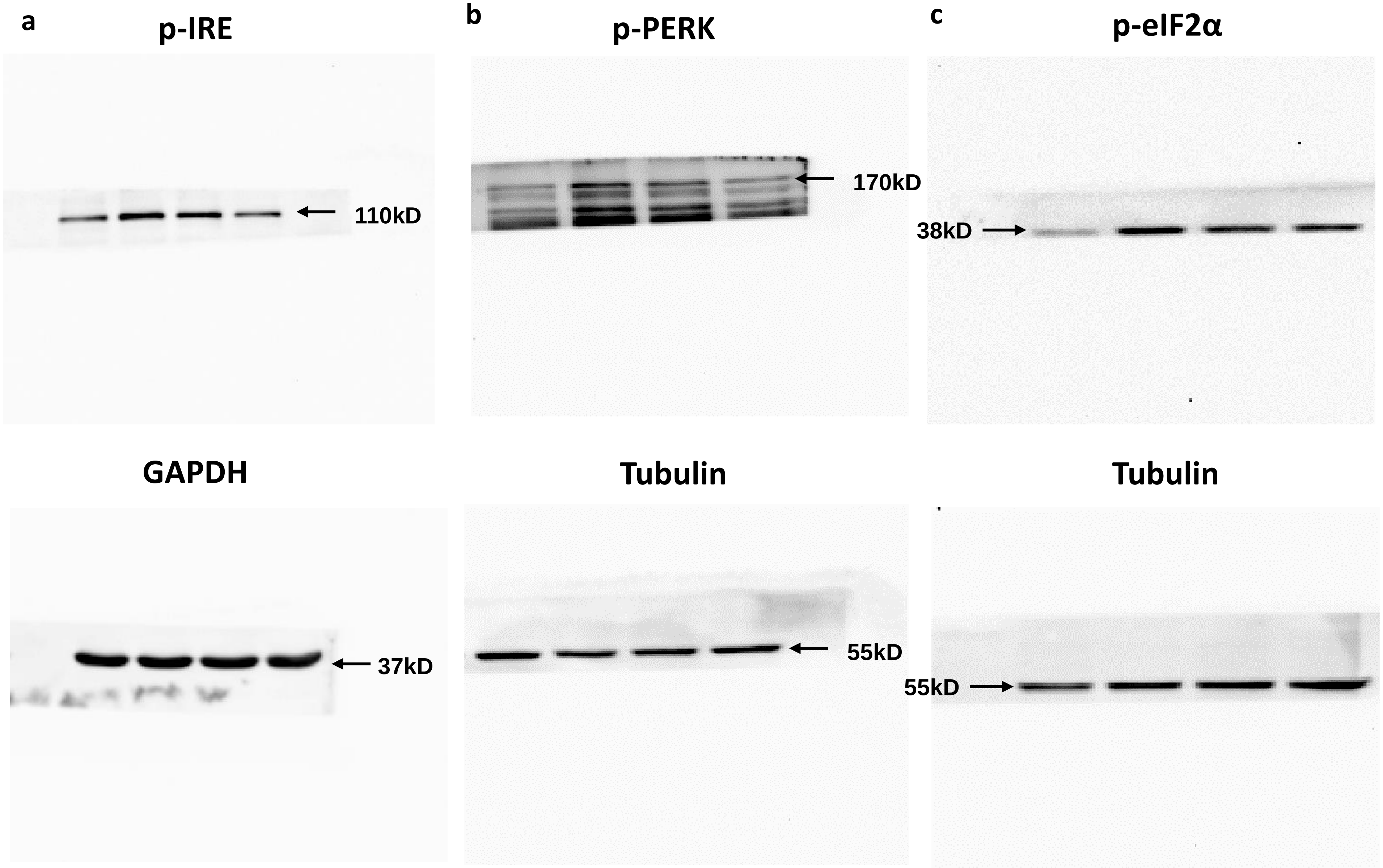

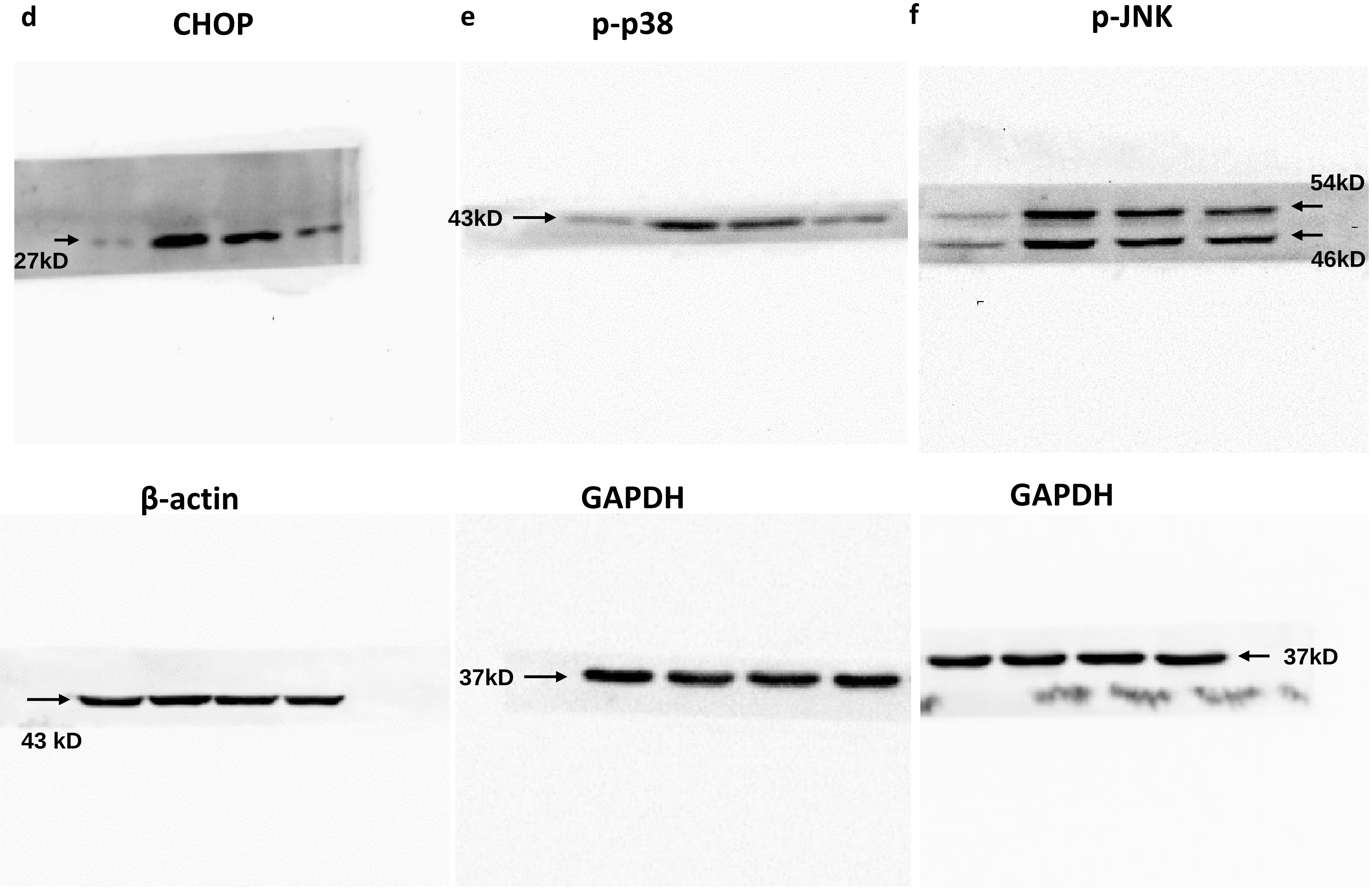
d
CHOP
27kD
e
p-p38
43kD
f
p-JNK
54kD
46kD
β-actin
43 kD
GAPDH
37kD
GAPDH
37kD

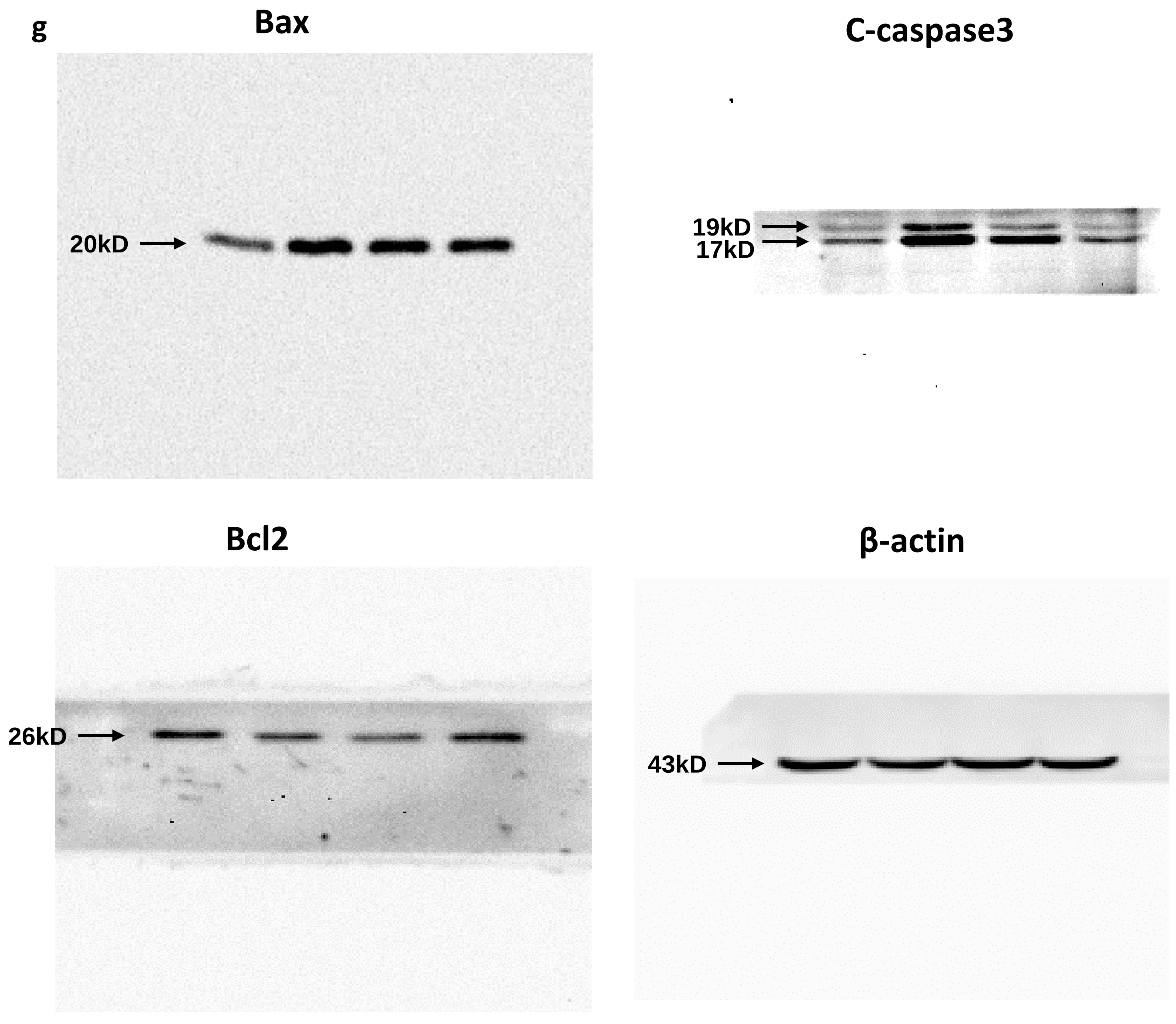
g
Bax
20kD
C-caspase3
19kD
17kD
Bcl2
26kD
β-actin
43kD

h

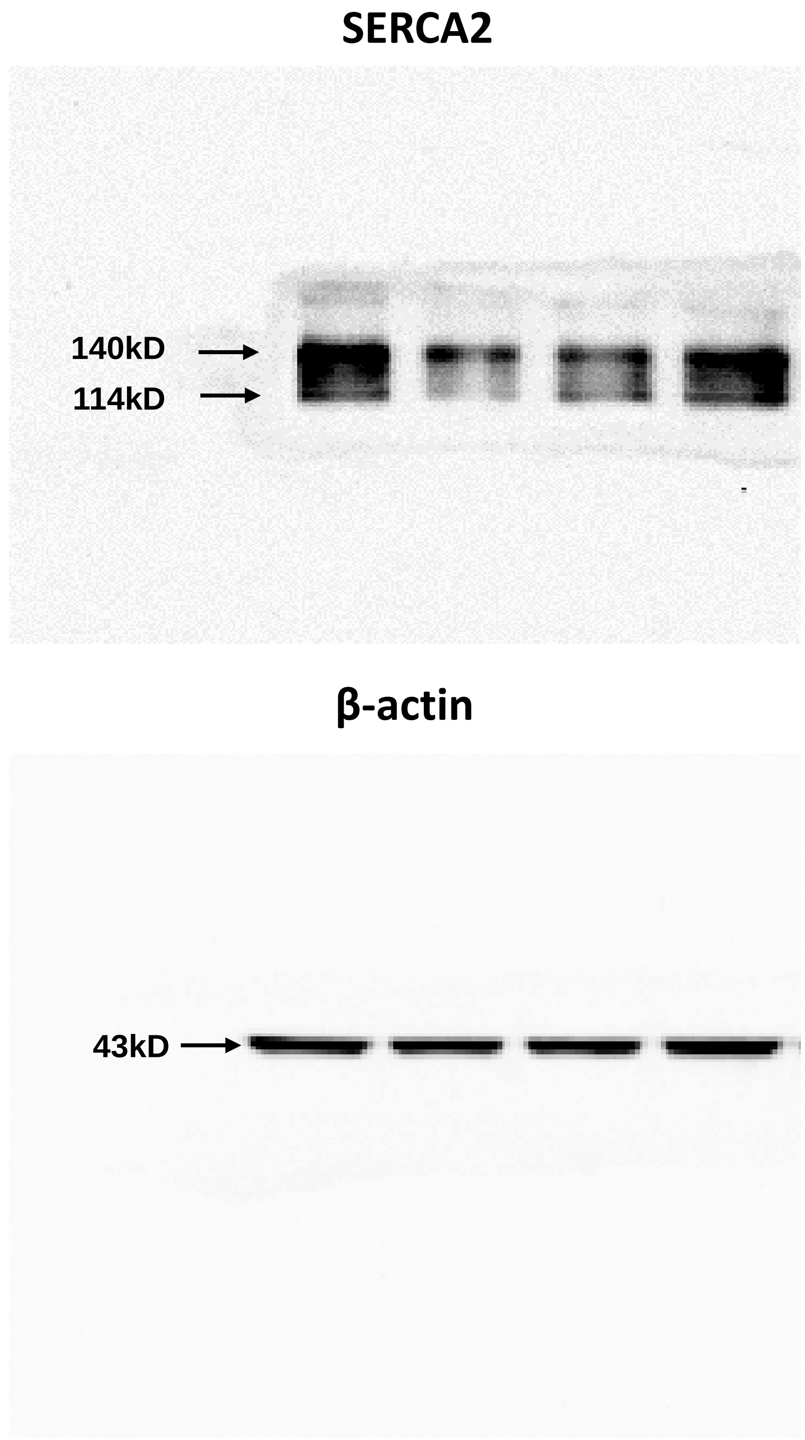

i

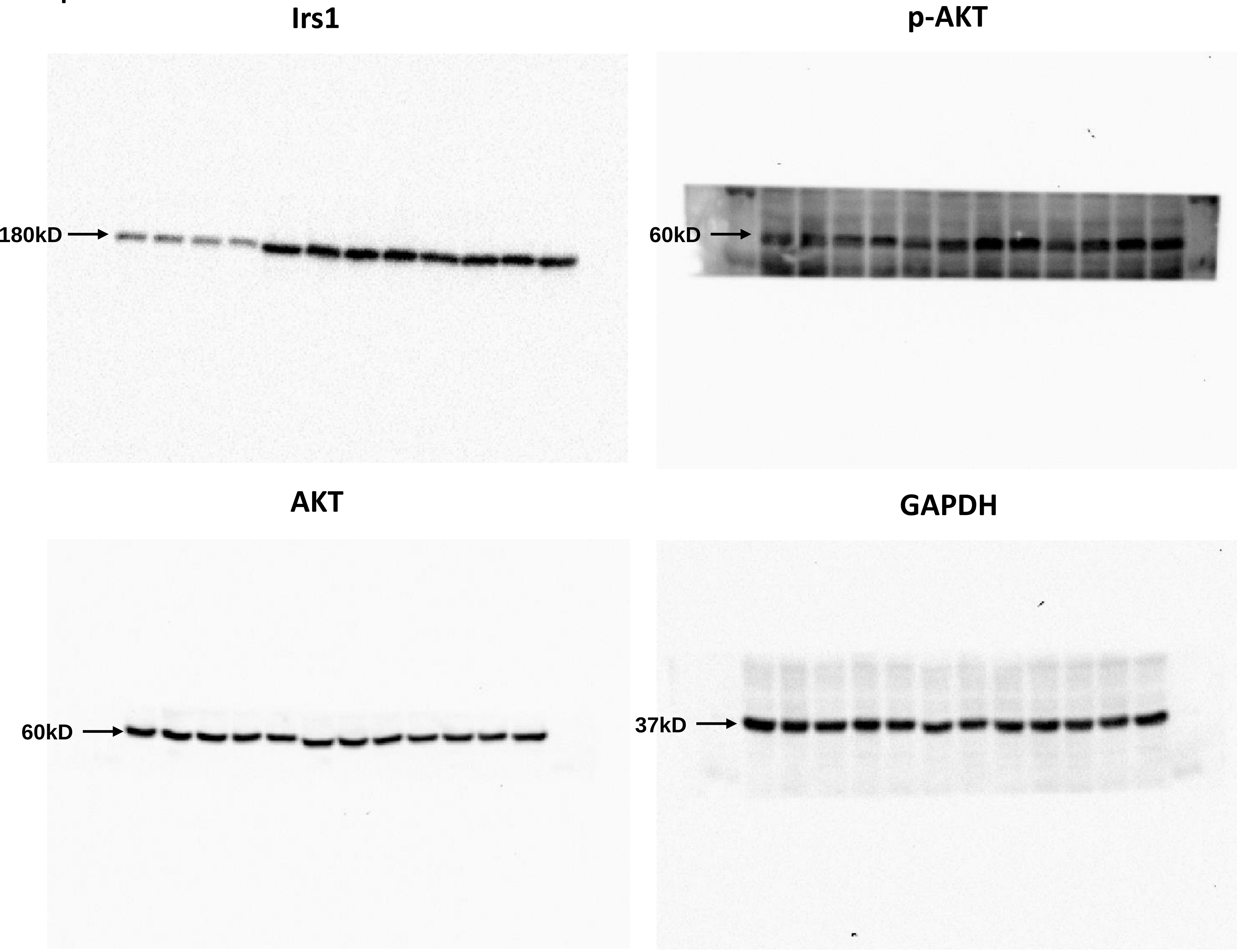

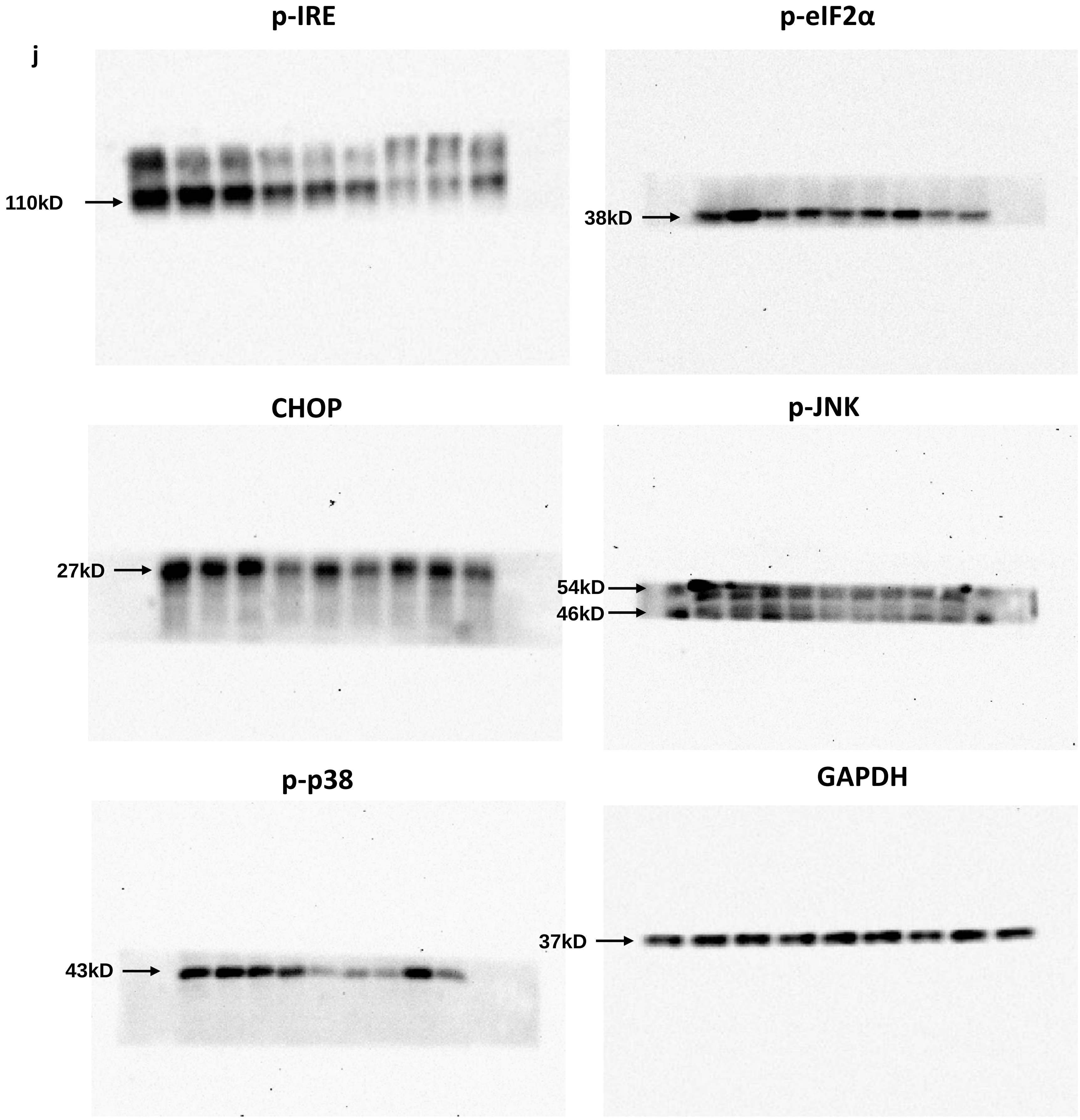

j
p-IRE
110kD
p-eIF2α
38kD
CHOP
27kD
p-JNK
54kD
46kD
p-p38
43kD
GAPDH
37kD

k

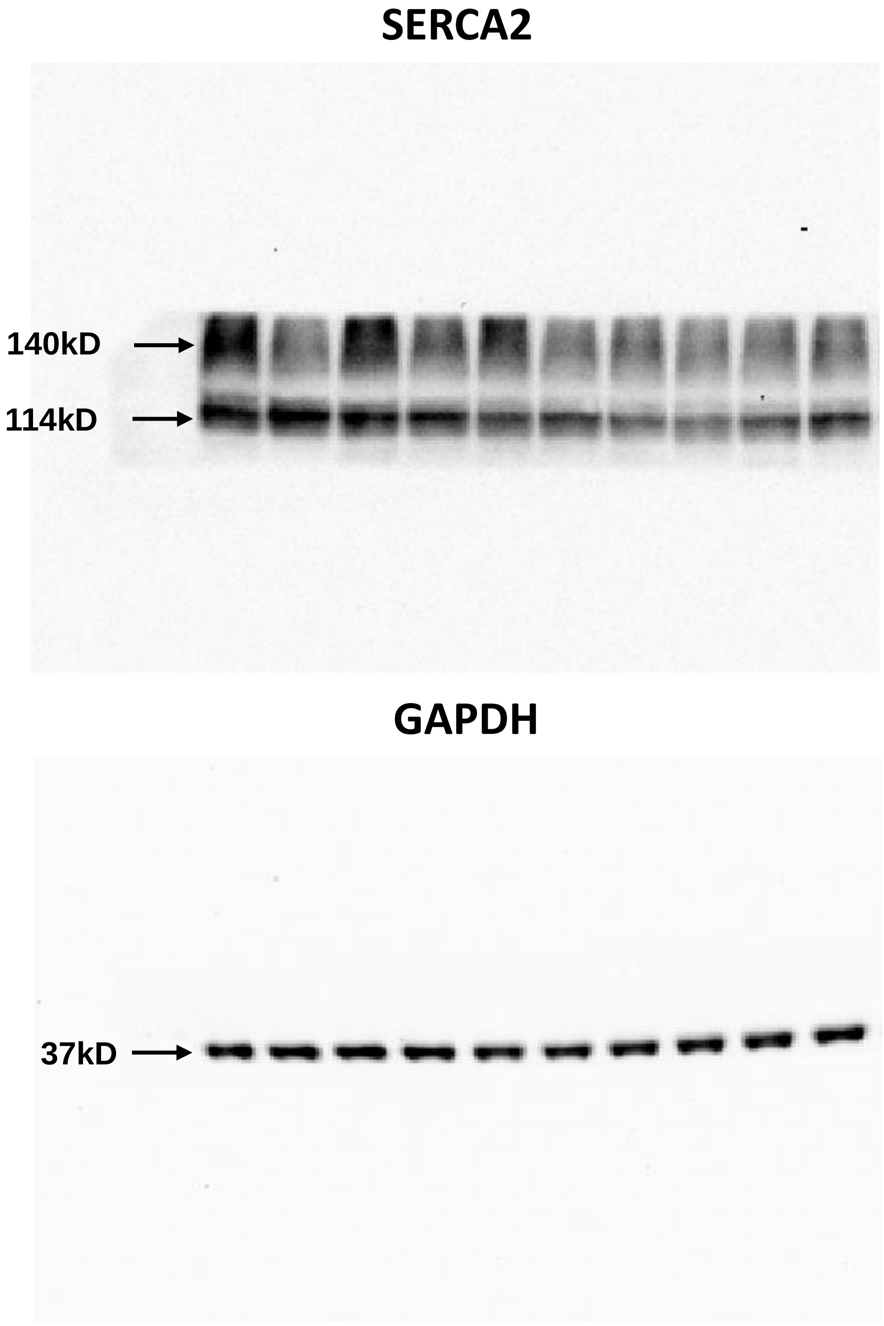

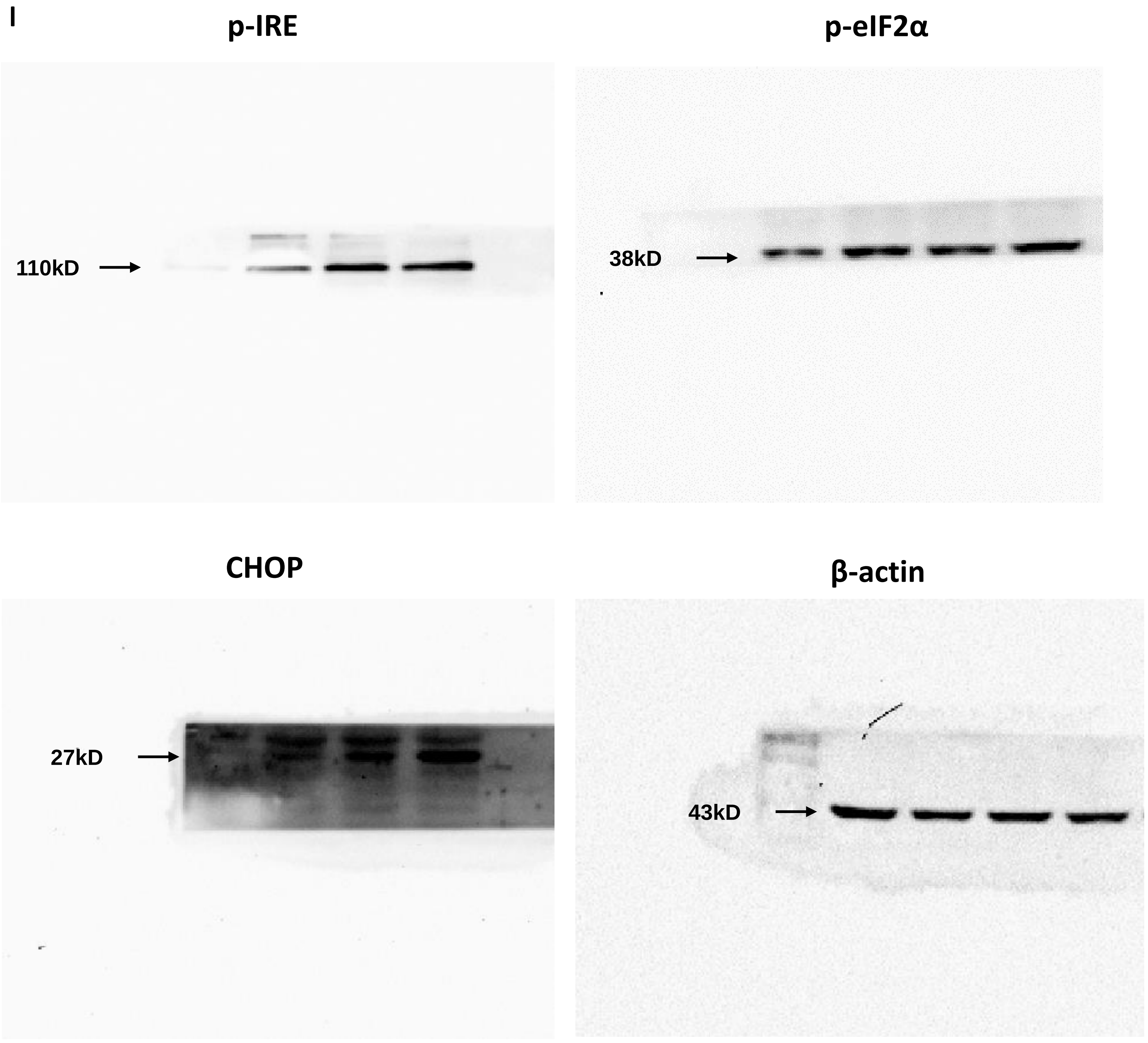
I
p-IRE
110kD
p-eIF2α
38kD
CHOP
27kD
β-actin
43kD

Supplementary Figure 5. Full western blot gel panels. **(a, b, c, d, e, f)** from which the data in **Figure 3A-F** were derived. **(g)** from which the data in **Figure 4A** were derived. **(h)** from which the data in **Figure 5E** were derived.**(i)** from which the data in **Figure 6I** were derived .**(j)** from which the data in **Figure 8A** were derived. **(k)** from which the data in **Figure 8C** were derived. **(l)** from which the data in **Supplementary Figure 3A** were derived.

SUPPLEMENTAL MATERIAL

Supplementary Table 1. Sequences of the real time PCR primers used in this study

Primers	Forward	Reverse
GAPDH(human)	CTCAGACACCAGGGGAAGGTGA	ATGATCTTGAGG CTGTTGTCATA
GAPDH	AAGGTCATCCCAGAGCTGAA	CTGCTTCACCACCTTCTTGA
CHOP	CAGATTCCAGTCAGAGTTCTAT GG	GACCACTCTGTTTCCGTTTCCT
ATF4	CCTCTGGACAAGGGTTTTGTG	GCACCGTGAAGACATCCCC
sXBP1	GCCATCAAGGACGTCAGCA	CTTCCTCCGAGTAGCGAATCAG
Bip	TCTGCAGGTCGTCCGACTATTC	AGGCAGTGGCTGTGAGATGC
Abcc3	ACGGACTTTATCATTGTGCT	ATCTGGTGCGTAGTTTCG
Abcc11	CACCATGCCCTCTACAA	CACCATCGCAAGGAACT
Cyp7a1	ACAGAAGCATAGACCCAA	TGCCAAACAGCGTTAGAT
SERCA2(human)	GATATGCTCATGTGGTGTTG	AATCTTCTTCAGTCGCTCCA
RyR	TGGGATTACAGGAAGACG	ATCGCTCAGAGGAGACACT
